# Structural Peculiarities and Thermoelectric Study of Iron Indium Thiospinel

**DOI:** 10.1002/chem.201905665

**Published:** 2020-04-06

**Authors:** Paweł Wyżga, Igor Veremchuk, Matej Bobnar, Primož Koželj, Steffen Klenner, Rainer Pöttgen, Andreas Leithe‐Jasper, Roman Gumeniuk

**Affiliations:** ^1^ Institut für Experimentelle Physik TU Bergakademie Freiberg Leipziger Strasse 23 09599 Freiberg Germany; ^2^ Max-Planck-Institut für Chemische Physik fester Stoffe Nöthnitzer Strasse 40 01187 Dresden Germany; ^3^ Institut für Anorganische und Analytische Chemie Universität Münster Corrensstrasse 3 48149 Münster Germany

**Keywords:** indium thiospinels, iron, phase diagrams, spinel phases, thermoelectric properties

## Abstract

The homogeneity range of ternary iron indium thiospinel at 873 K was investigated. A detailed study was focused on two distinct series (*y*=*z*): 1) a previously reported charge‐balanced (In_0.67+0.33*y*_□_0.33−0.33*y*_)_tetr_[In_2−*z*_Fe_*z*_]_oct_S_4_ (A1‐series; □ stands for vacancy; the abbreviations “tetr” and “oct” indicate atoms occupying tetrahedral 8*a* and octahedral 16*d* sites, respectively) and 2) a new charge‐unbalanced (In_0.67+*y*_□_0.33−*y*_)_tetr_[In_2−*z*_Fe_*z*_]_oct_S_4_ (A2‐series). Fe atoms were confirmed to exclusively occupy an octahedral position in both series. An unusual reduction of the unit cell parameter with increasing Fe content is explained by differences in the ionic radii between Fe and In, as well as by an additional electrostatic attraction originating from charge imbalance (latter only in A2‐series). The studied compound is an *n*‐type semiconductor, and its charge carrier concentration increases or decreases for larger Fe content within the A1‐ and A2‐series, respectively. The thermal conductivity *κ*_tot_ is significantly reduced upon increasing vacancy concentration, whereas the change of power factor is insufficient to drastically improve the thermoelectric figure of merit.

## Graphical abstract

**Iron indium thiospinel**: Two different mechanisms for the Fe incorporation into indium thiospinel were investigated, namely the charge‐balanced (A1) and the charge‐unbalanced (A2) series. The Fe content (i.e., vacancy concentration) strongly influences the unit cell parameter, charge carrier concentration, charge carrier mobility, as well as thermal conductivity (*κ*_tot_) in both series.

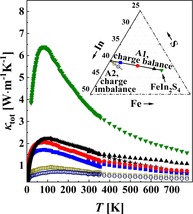

## Introduction

**Figure 1:**
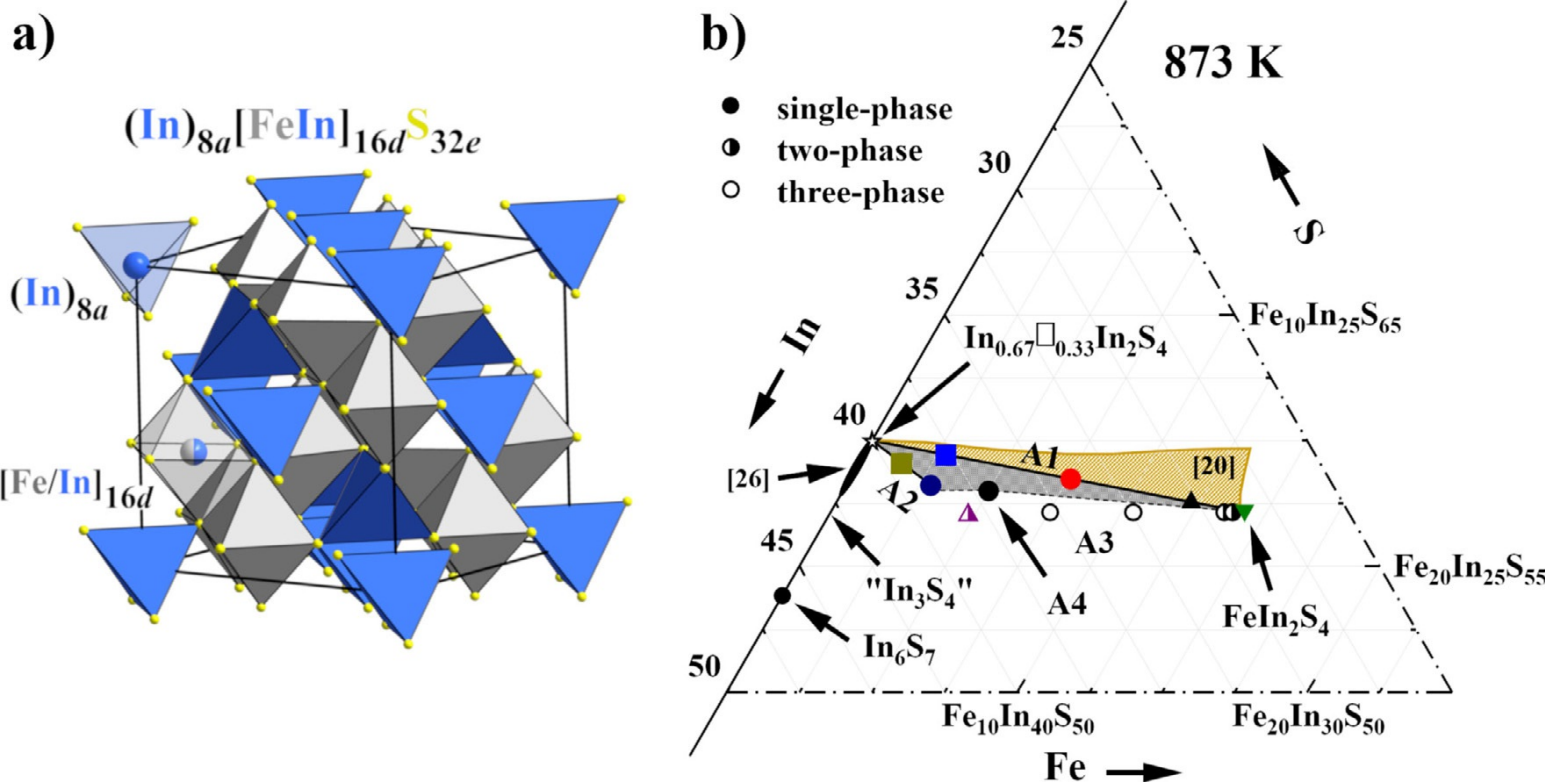
a) Crystal structure of (In)[FeIn]S_4_ thiospinel: space group *Fd* $\bar{3}$ *m*, *a*=10.6134(1) Å. The 16*d* position (centers of octahedral voids) is occupied by a statistical mixture of Fe/In atoms, whereas tetrahedra with the centers at the 8*a* site are exclusively filled by In atoms. For better visualization, Origin Choice 1 for space group *Fd* $\bar{3}$ *m* was chosen. b) Investigated part of the ternary phase diagram of the Fe‐In‐S system at 873 K based on powder X‐ray diffraction (PXRD) analysis. The proposed homogeneity range of iron indium thiospinel is shaded in gray, and the complementary region studied previously^20^ at 1073 K is marked in gold. Color code for the A1 and A2 samples is unified in all figures.

Spinel compounds adopt the MgAl_2_O_4_‐type structure (space group *Fd* $\bar{3}$ *m*, *a*≈9–11 Å), in which cations occupy one‐eighth of tetrahedral (Wyckoff position 8*a*) and half of the octahedral (16*d*) voids within nearly cubic‐close‐packed arrays of anions (O^2−^, S^2−^, Se^2−^, or Te^2−^; Figure 1 a).^1^ The spinel structure is known to be flexible and allows an accommodation of 1) a variety of metallic elements with different atomic size and mass, 2) ordered or disordered arrangement of cations (so‐called normal, inverse, or intermediate structures), and 3) partially occupied crystallographic positions. All these result in a diversity of magnetic,^2^, ^3^, ^4^ optical,^5^ catalytic,^6^ electrical,^7^, ^8^, ^9^ as well as thermoelectric (TE) properties.^10^, ^11^, ^12^, ^13^, ^14^ Many spinels consisting of well‐abundant and environmental‐friendly elements (which is crucial for a possible thermoelectric application) became the object of numerous studies. Among these compounds, indium‐based thiospinels are a rare case of *n*‐type sulfide semiconductors.^15^

Recent studies^16^, ^17^ revealed that even the binary In_1−*x*_□_*x*_In_2_S_4_ (*x*=0.33) thiospinel (□ stands for vacancy) presents a promising TE figure of merit *ZT* between 0.2–0.4 above 700 K. The further incorporation of In in the compound (i.e., *x*=0.22 and 0.16) is accompanied with an enhancement of structural disorder (which promotes an intrinsically low thermal conductivity *κ*_tot_<0.5 W m^−1^ K^−1^ above RT), as well as with a reduction of the charge carrier concentration (*n*<10^18^ cm^−3^ at 298 K) leading to a decrease of the *ZT* parameter.^16^ Seemingly, the simplest way to improve TE efficiency (i.e., to enhance *n*) of the In_1−*x*_□_*x*_In_2_S_4_ thiospinel would be an incorporation of the third element into its structure.

One of the most studied ternary indium thiospinels is FeIn_2_S_4_. It is an example of a so‐called nearly inverse spinel, in which Fe^2+^ and half of In^3+^ ions occupy octahedral interstices and the rest of In fills tetrahedral voids.^18^, ^19^, ^20^ Crystallographic positions of Fe and In are in agreement with their site preferences discussed by Busch and Hulliger.^21^ A continuous solid solution is reported for the In_0.67_□_0.33_In_2_S_4_–FeIn_2_S_4_ 20, 22, 23, 24 cross‐section, which allows tuning of the physical properties of this thiospinel by changing the Fe concentration.

There is hardly any information about the thermoelectric properties of iron indium thiospinel. The electrical conductivity *σ* was measured for the In_0.67_□_0.33_In_2_S_4_–FeIn_2_S_4_ solid solution, but no trend of *σ* versus chemical composition was found.^22^ The influence of Fe content on electronic and thermal transport properties remains unclear. Interestingly, a linear decrease of the direct energy gap with increase of Fe content is reported in the series,^24^ which would suggest a possible enhancement of charge carrier concentration of In_0.67_□_0.33_In_2_S_4_ by Fe‐for‐In substitution. To verify this concept as well as to check the influence of Fe incorporation into the In_0.67_□_0.33_In_2_S_4_ structure on its TE properties, we performed a systematic investigation of iron indium thiospinel. This study deals with compounds crystallizing with the spinel‐type structure within the In_0.67_□_0.33_In_2_S_4_–FeIn_2_S_4_ solid solution (A1 series), as well as within the In_0.67_□_0.33_In_2_S_4_–Fe (A2) and the “InIn_2_S_4_”–FeIn_2_S_4_ (A3) cross‐sections in the ternary Fe‐In‐S system (Figure 1 b). Structural peculiarities as well as magnetic and thermoelectric properties are discussed as a function of temperature and Fe content/vacancy concentration.

## Results and Discussion

### Homogeneity range of iron indium thiospinel

**Figure 2:**
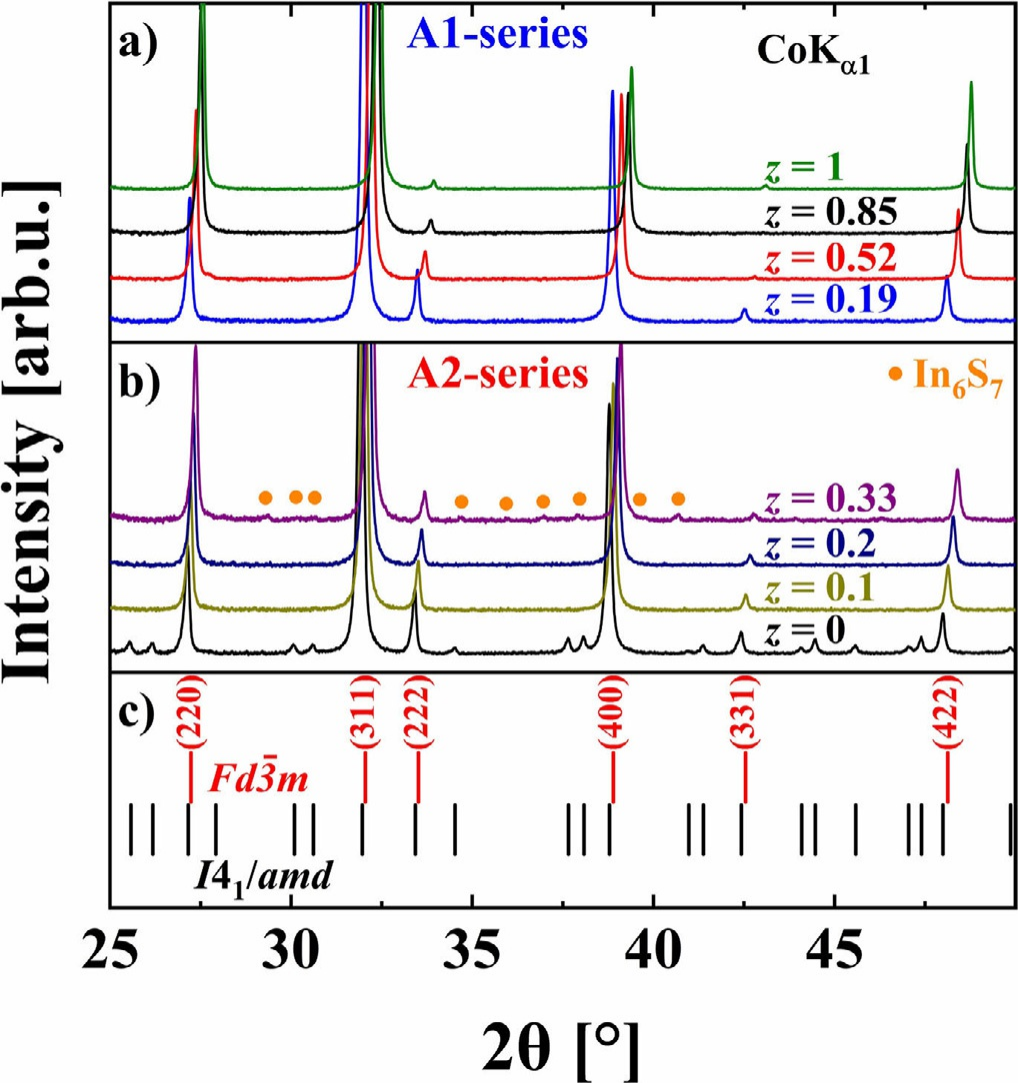
PXRD patterns of Fe‐In‐S samples from the series a) A1 and b) A2 together with c) the theoretical positions of reflections for tetragonal β‐In_0.67_□_0.33_In_2_S_4_ (*I*4_1_/*amd*) and cubic MgAl_2_O_4_ (*Fd* $\bar{3}$ *m*) type structures. Intensities within each pattern were normalized according to the most intense reflection.

To investigate the homogeneity range of the ternary iron indium thiospinel at 873 K, four series of samples were synthesized (Figure 1 b and Table 1). These series are given with formulas (In_0.67+0.33*y*_□_0.33−0.33*y*_)_tetr_[In_2−*z*_Fe_*z*_]_oct_S_4_ (labeled as A1), (In_0.67+*y*_□_0.33−*y*_)_tetr_[In_2−*z*_Fe_*z*_]_oct_S_4_ (A2), and (In)_tetr_[In_2−*z*_Fe_*z*_]_oct_S_4_ (A3) (the abbreviations “tetr” and “oct” indicate atoms occupying tetrahedral 8*a* and octahedral 16*d* sites, respectively), as well as a separate composition Fe_0.345_In_2.552_S_4_ (A4). The PXRD phase analysis (Figure 2 and Figure S1a, Supporting Information) confirms the samples from the series A1 (0≤*y*=*z*≤1), A2 (0≤*y*=*z*≤0.2), and A4 to be single phase, whereas the other specimens from the series A2 (*z*=0.33) and A3 contain secondary phases (Figure S1, Supporting Information). These findings are confirmed by optical metallography and energy‐dispersive X‐ray spectroscopy (EDXS) analysis (Figure S2 and S3, Supporting Information). SEM/EDXS revealed also some small inclusions of Fe_1−*x*_S in the above‐mentioned single‐phase materials. They are below the detection limit of PXRD and thus are neglected in the further discussions of the physical properties. Experimentally obtained chemical compositions from wavelength‐dispersive X‐ray spectroscopy (WDXS) of the studied samples are in good agreement with the nominal ones (Table 1). On the basis of these results, we propose a homogeneity range of iron indium thiospinel at 873 K (shaded in gray in Figure 1 b), which complements the previously reported region at 1073 K^20^ (gold in Figure 1 b).

**Table 1:** Chemical and phase composition as well as applied heat treatment for the studied series: A1: (In_0.67+0.33*y*_□_0.33−0.33*y*_)_tetr_[In_2−*z*_Fe_*z*_]_oct_S_4_, A2: (In_0.67+*y*_□_0.33−*y*_)_tetr_[In_2−*z*_Fe_*z*_]_oct_S_4_, and A3: (In)_tetr_[In_2−*z*_Fe_*z*_]_oct_S_4_, as well as for the Fe_0.345_In_2.552_S_4_ composition. To emphasize that Fe occupies the 16*d* position only, we introduced an individual index *z*=*y*.

Series	*z*	Chemical composition		Phases detected after last annealing	Heat treatment^[b]^
		nominal	WDXS^[a]^		
A1	0	In_2.67_S_4_	In_2.66(1)_S_4.00(1)_	β‐In_0.67_□_0.33_In_2_S_4_	refer to 16
	0.19	Fe_0.19_In_2.54_S_4_	Fe_0.18(1)_In_2.56(1)_S_4.00(2)_	spinel	a
	0.52	Fe_0.52_In_2.32_S_4_	Fe_0.52(1)_In_2.34(1)_S_4.00(2)_	spinel	b, a
	0.85	Fe_0.85_In_2.1_S_4_	Fe_0.84(2)_In_2.12(2)_S_4.00(1)_	spinel	c, a
	1	FeIn_2_S_4_	Fe_0.99(1)_In_2.02(1)_S_4.00(1)_	spinel	b, a
					
A2	0.1	Fe_0.1_In_2.67_S_4_	Fe_0.10(1)_In_2.73(2)_S_4.00(2)_	spinel	a
	0.2	Fe_0.2_In_2.67_S_4_	Fe_0.19(1)_In_2.71(2)_S_4.00(2)_	spinel	a
	0.33	Fe_0.33_In_2.67_S_4_	Fe_0.36(1)_In_2.62(2)_S_4.00(1)_	spinel+In_6_S_7_	a
					
A3	0.53	Fe_0.53_In_2.47_S_4_	–	2 spinels+FeS+In_6_S_7_	a
	0.73	Fe_0.73_In_2.27_S_4_	–	2 spinels+FeS+In_6_S_7_	a
	0.95	Fe_0.95_In_2.05_S_4_	–	spinel+In_6_S_7_	b, a
	0.97	Fe_0.97_In_2.03_S_4_	–	spinel+In_6_S_7_	b, a
					
A4	0.345	Fe_0.345_In_2.552_S_4_	–	spinel	a

[a] Chemical compositions were normalized assuming full occupancy of the S site (32*e*) in the spinel structure. [b] Annealing conditions: a: 873 K/168 h+quenching in liquid nitrogen; b: 1073 K/336 h+quenching in liquid nitrogen; c: 1073 K/72 h+slow cooling (approximately 66 K h^−1^).

**Figure 3:**
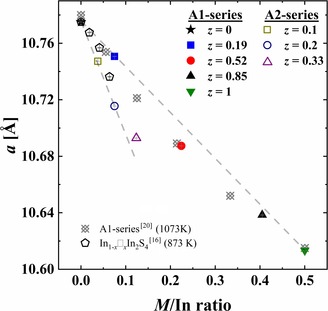
Unit cell parameters (UCPs) versus *M*/In ratio of samples from the A1‐ and A2‐series after synthesis as well as previously reported data.^16^, ^20^ *M*=Fe or In (excess of In in comparison with composition In_0.67_□_0.33_In_2_S_4_, only for binary samples^16^). Numbers indicate the Fe content per respective formula unit. Error bars (in gray) are smaller than chosen symbols .

PXRD phase analysis clearly indicates that incorporation of a small amount of iron into the initially tetragonal In_0.67_□_0.33_In_2_S_4_ (the β polymorph)^16^ leads to a stabilization of the cubic MgAl_2_O_4_ structure type (*Fd* $\bar{3}$ *m*, *a*≈10 Å, Figure 1 a) for the studied thiospinels. The unit cell parameter (UCP) decreases monotonously for larger Fe content (lower vacancy concentration) in both sample series (in agreement with the previous report,^20^ Figure 3). However, the decrease of UCPs for the single‐phase A2 samples is steeper. No changes in UCPs for both series were observed after spark plasma sintering (SPS) and high‐temperature (HT) transport measurements (LFA, ZEM‐3), thus indicating the thermal stability of the materials. Reduction of UCP for lower vacancy concentration, observed also for binary In_1−*x*_□_*x*_In_2_S_4_ thiospinel,^16^, ^25^, ^26^ is an unusual effect and there is no solid explanation for it so far.

### Structural peculiarities of iron indium thiospinels

The reduction of UCP was also found for other *M_x_*In_*y*_S_4_ (*M*=Co,^27^ Ni,^27^ Cu^28^) thiospinels. On the other hand, compounds containing *M*=Na,^29^ Ag^30^, and Cd^31^ reveal an opposite trend (i.e., increase of UCPs with incorporation of the 3^rd^ element), which is comprehensible, taking into account that fillers have to occupy the former vacancies (Figure 1 and Figure S4, Supporting Information). Interestingly, important observations can be made for these three cases: 1) in the reported structures^29^, ^30^, ^31^ *M* atoms always occupy 8*a* sites, 2) ionic radii of filler elements such as *r* ${}_{\mathrm{Na}{}^{+}}$ =0.99 Å, *r* ${}_{\mathrm{Ag}{}^{+}}$ =1 Å, and *r* ${}_{\mathrm{Cd}{}^{2+}}$ =0.78 Å [for tetrahedral coordination, coordination number (CN)=4] are notably larger than that of In (*r* ${}_{\mathrm{In}{}^{3+}}$ =0.62 Å),^32^ 3) metal–sulfur interatomic distances within the tetrahedra (*d*_[*M*(8*a*)−S]_) increase in comparison to those in the binary In_0.67_□_0.33_In_2_S_4_, and 4) no change of metal–sulfur contacts (*d*_[*M*(16*d*)−S]_) is observed within the octahedra (Table 2). The first three tendencies are exactly opposite for the *M_x_*In_*y*_S_4_ (*M*=Co, Ni, Cu) thiospinels, in which 1) *M* atoms occupy the 16*d* position (with the exception for Cu, which is in 8*a*), 2) ionic radii *r* ${}_{\mathrm{Co}{}^{2+}}$ =0.745 Å, *r* ${}_{\mathrm{Ni}{}^{2+}}$ =0.69 Å (CN=6) and *r* ${}_{\mathrm{Cu}{}^{+}}$ =0.6 Å (CN=4) are smaller than *r* ${}_{\mathrm{In}{}^{3+}}$ =0.8 Å (CN=6), 0.62 Å (CN=4), and 3) metal–sulfur interatomic distances *d*_[*M*(16*d*)−S]_ within the octahedra are reduced (*d*_[*M*(8*a*)−S]_ for the Cu compound were not available). Apparently, the different distribution of the *M* cations (i.e., over tetrahedral or/and octahedral sites) and consequently their ionic radii determine the observed trends of UCP in these thiospinels. Therefore, in further steps of our analysis we paid special attention to the crystallographic distribution of Fe in the structures of the A1‐ and A2‐series.

**Table 2:** Comparison of metal–sulfur interatomic distances within tetrahedra *d*_[*M*(8*a*)−S]_ and octahedra *d*_[*M*(16*d*)−S]_. Δ stands for a relative change of the distance with respect to the In_0.67_□_0.33_In_2_S_4_ sample.

Series	Nominal composition	*d*_[*M*(8*a*)−S]_ [Å]/Δ [%]	*d*_[*M*(16*d*)−S]_ [Å]/Δ [%]
In_1−*x*_□_*x*_In_2_S_4_ 16	In_0.67_□_0.33_In_2_S_4_^[a]^	2.46(1)/–	2.63(1)/–
			
Ag‐In‐S (Ag at 8*a*)^30^	Ag_0.5_In_2.5_S_4_	2.496/+1.1	2.622/≈0
Na‐In‐S (Na at 8*a*)^29^	Na_0.5_In_2.5_S_4_	2.511/ +1.7	2.616/≈0
			
Co‐In‐S (Co mainly at 16*d*)^27^	CoIn_2_S_4_	2.453(3)/≈0	2.553(3)/−4
Ni‐In‐S (Ni at 16*d*)^27^	NiIn_2_S_4_	2.458(4)/≈0	2.525(4)/−4
			
A1‐series (Fe at 16*d*)	(In_0.84_□_0.16_)[Fe_0.52_In_1.48_]S_4_	2.450(6)/≈0	2.593(6)/−1.4
	(In_0.95_□_0.05_)[Fe_0.85_In_1.15_]S_4_	2.457(7)/≈0	2.571(7)/−2.2
	(In)[FeIn]S_4_	2.455(5)/≈0	2.564(5)/−2.5
			
In_1−*x*_□_*x*_In_2_S_4_ 16	In_0.78_□_0.22_In_2_S_4_	2.452(3)/−0.7	2.621(3)/≈0
	In_0.84_□_0.16_In_2_S_4_	2.435(3)/−1.4	2.622(3)/≈0

[a] High‐resolution (HR) PXRD pattern was refined assuming space group *Fd* $\bar{3}$ *m*, instead of *I*4_1_/*amd*.

To this end we analyzed the intensities of the (220) and (222) reflections, given that they are exclusively related to the site occupancy factors (SOFs) of the 8*a* and 16*d* Wyckoff positions, respectively.^33^ As one can see in the experimental PXRD patterns (Figure 2), the intensities of the (220) and (222) reflections increase and decrease, respectively, for higher Fe content. To compare data for different samples, the background was subtracted from the PXRD data and then the intensity was normalized according to the most intense peak. The theoretical patterns were simulated for two scenarios (Table 3): 1) Fe atoms fill the 8*a* (tetrahedral) site in a statistical mixture with In and vacancies, whereas the octahedral 16*d* position is fully occupied by In (Scenario 1), and 2) the 8*a* site is partially filled by In, whereas octahedra (16*d* position) are occupied with a statistical mixture of Fe and In (Scenario 2). As one can see from Table 3, the same increase of the Fe‐content *z* results in a stronger reduction of the vacancy concentration in the case of the A2‐series.

**Table 3:** Scenarios of Fe‐incorporation into (In_0.67_□_0.33_)_tetr_[In_2_]_oct_S_4_.^[a, b, c, d]^

Sce‐	A1‐series (charge balance)		A2‐series (charge imbalance)	
nario	8*a*	16*d*	8*a*	16*d*
1	*z* Fe+(In_0.67_□_0.33_) ↓ (Fe_*z*_In_0.67−0.67*z*_□_0.33−0.33*z*_)	no change [In_2_]	*z* Fe+(In_0.67_□_0.33_) ↓ (Fe_*z*_In_0.67_□_0.33−*z*_)	no change [In_2_]
				
2	0.33*y* In+(In_0.67_□_0.33_) ↓ (In_0.67+0.33*y*_□_0.33−0.33*y*_)	*z* Fe+[In_2_] ↓ [Fe_*z*_In_2−*y*_]+0.33*y* In	*y* In+(In_0.67_□_0.33_) ↓ (In_0.67+*y*_□_0.33−*y*_)	*z* Fe+[In_2_] ↓ [Fe_*z*_In_2−*y*_] +*y* In

[a] On the basis of observed effective magnetic moments, Fe^2+^ ions were assumed. [b] Octahedral site (16*d*) is always fully occupied (SOF=1). [c] To keep the charge balance in the A1‐series, the Fe‐for‐In substitution ratio is equal to 3:2. [d] *y*=*z* (see Table 1 and discussion).

**Figure 4:**
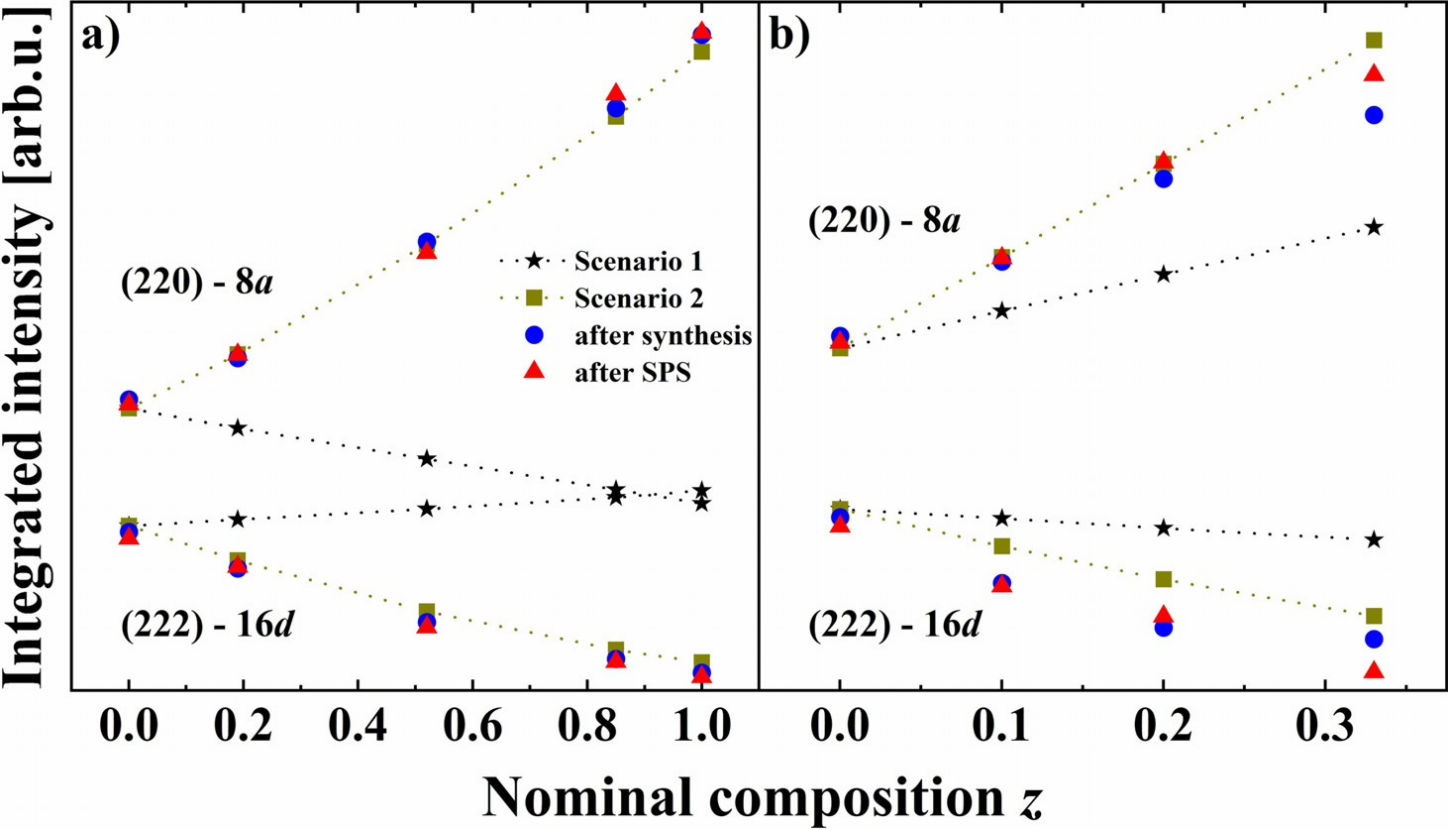
Evolution of integrated intensity versus nominal Fe content *z* for (220) and (222) reflections, exclusively related to the tetrahedral 8*a* and octahedral 16*d* Wyckoff positions, respectively. a) A1‐series (In_0.67+0.33*y*_□_0.33−0.33*y*_)_tetr_[In_2−*z*_Fe_*z*_]_oct_S_4_ (charge balance) and b) A2‐series (In_0.67+*y*_□_0.33−*y*_)_tetr_[In_2−*z*_Fe_*z*_]_oct_S_4_ (charge imbalance). Scatter plot indicates experimental data, and line–scatter plot indicates theoretical data. Scenario 1: Fe at the 8*a* site; Scenario 2: Fe at the 16*d* site (see details in text). The color code is the same in both figures.

Both observed and theoretical (calculated for Scenario 1 and 2) intensities are compared in Figure 4. For the A1‐series, experimental intensities (including those after SPS treatment) perfectly coincide with the values calculated assuming Scenario 2 (Figure 4 a). This could indicate a substitution of In by Fe at the 16*d* sites and would be in agreement with previous structural studies.^19^, ^20^, ^23^ For the A2‐series, a similar trend is observed: Scenario 1 can be excluded, whereas Scenario 2 provides an acceptable agreement between experimental and theoretical intensities for both (220) and (222) reflections (Figure 4 b).

As a result of the small number of observed reflections (Table S1, Figure S5, Supporting Information), a Rietveld refinement could be performed only for the samples with the highest Fe content (*z≥*0.52). In accordance with the analysis performed above, Fe atoms were assumed to occupy the octahedral voids in the starting model. The refinement converged for all three specimens with low reliability factors, and the refined chemical compositions were in good agreement with those obtained from WDXS (crystallographic data and atomic parameters are summarized in Table S1, Supporting Information). The performed refinements confirmed the correctness of the previous conclusion about the occupancies of octahedral and tetrahedral voids in the studied thiospinel.

Knowing that Fe atoms are incorporated exclusively in octahedra and that they have a +2 oxidation state (see Magnetic susceptibility section, Table 4), one could speculate on the reasons for the unexpected decrease of UCP in the A1‐ and A2‐series with increasing Fe content. To model such an effect, one would need to start with the binary In−S thiospinel. Assuming In ions have a +3 oxidation state, one immediately recognizes that the only charge‐balanced composition within the In_1−*x*_□_*x*_In_2_S_4_ series would be the one with *x*=0.33. Further decrease of *x* (i.e., incorporation of additional In atoms in the structure) leads to a charge imbalance in this thiospinel. The charge imbalance in turn can cause additional electrostatic attractions between In^3+^ and S^2−^ ions within the tetrahedra, which then results in a shortening of In−S contacts (Table 2) and thus in the decrease of UCPs.^16^ A similar phenomenon was observed for ZrO_2−*x*_ and CeO_2−*x*_.^34^

**Table 4:** Magnetic parameters of (In_0.67+0.33*y*_)[In_2−*z*_Fe_*z*_]S_4_ (A1) and (In_0.67+*y*_)[In_2−*z*_Fe_*z*_]S_4_ (A2) samples (*y*=*z*).

Series	*z*	Ordering^[a]^	*T*_N_ [K]^[b]^	*θ*_CW_ [K]	*μ*_eff_ [*μ*_B_]^[c]^	*μ*_eff_ (literature)
A1	0.19	n.d.	n.d.	−16.8(3)	4.62	–
	0.52	AF	4.7	−63.3(2)	5.2	–
	0.85	AF	8.7	−89.7(3)	5.11	–
	1	AF	11.4	−112.8(3)	5.28	5.3^37^, 4.98^38^
						
A2	0.1	n.d.	n.d.	−1.8(4)	4.76	–
	0.2	n.d.	n.d.	−22.4(2)	5.04	–
	0.33	n.d.	n.d.	−46.2(3)	4.54	–

[a] AF=antiferromagnetic, In_0.67_□_0.33_In_2_S_4_ (*z*=0) is diamagnetic, n.d.=not detected. [b] *T*_N_‐Néel temperature. [c] Values calculated for the Fe‐content according to WDXS.

The same consideration can be done for iron indium thiospinel. It is clearly visible that the common formula (In_0.67+0.33*y*_□_0.33−0.33*y*_)_tetr_[In_2−*z*_Fe_*z*_]_oct_S_4_ for the A1‐series assumes charge‐balanced compounds (under the condition that In ions have a +3 oxidation state), whereas the (In_0.67+*y*_□_0.33−*y*_)_tetr_[In_2−*z*_Fe_*z*_]_oct_S_4_) formula for the A2‐series indicates some charge imbalance (for *z*>0). From the discussion above, the only reason for the reduction of UCP with increasing Fe content for the A1‐series would be the substitution of larger In^3+^ (*r* ${}_{\mathrm{In}{}^{3+}}$ =0.8 Å, CN=6) by smaller Fe^2+^ (*r* ${}_{\mathrm{Fe}{}^{2+}}$ =0.78 Å, CN=6) in the structure (similar to that observed for indium thiospinels with Co, Ni, Cu, Table 2; see discussion above). On the other hand, the steeper decrease of UCPs for the A2‐series, in comparison with those of the A1‐samples (Figure 3), could be accounted for by additional effects due to charge imbalance.

To simplify the presentation of the chemical formulas, in the further text we skip vacancies and labels, that is, (In_0.67+0.33*y*_)[In_2−*z*_Fe_*z*_]S_4_ (*y*=*z*) instead of (In_0.67+0.33*y*_□_0.33−0.33*y*_)_tetr_[In_2−*z*_Fe_*z*_]_oct_S_4_.

### Mössbauer spectroscopy

The ^57^Fe Mössbauer spectra at 6 K for the samples with *z*=0.1, 0.2, 0.33 (A2‐series) and 0.52 (A1‐series) are presented in Figure S6 (Supporting Information). Despite the long counting times, the (In)[Fe_0.33_In_1.67_]S_4_ and (In_0.84_)[Fe_0.52_In_1.48_]S_4_ spectra showed only moderate signal‐to‐noise ratios and we obtained no satisfying fit. Therefore, only the experimental spectra for these samples are shown in Figure S6 (Supporting Information). The fitting parameters for the (In_0.77_)[Fe_0.1_In_1.9_]S_4_ and (In_0.87_)[Fe_0.2_In_1.8_]S_4_ spectra are summarized in Table S2 (Supporting Information). Both spectra were well‐reproduced with simple doublets. The isomer shift values are typical for octahedral high‐spin Fe^2+^ (*d* ^6^ configuration), similar to that of pure (In)[FeIn]S_4_.^20^, ^35^, ^36^ Within the resolution of the spectra, there was no hint of an additional spectral component. These observations are in good agreement with the performed analysis of structural peculiarities in the iron indium thiospinel.

### Magnetic susceptibility

**Figure 5:**
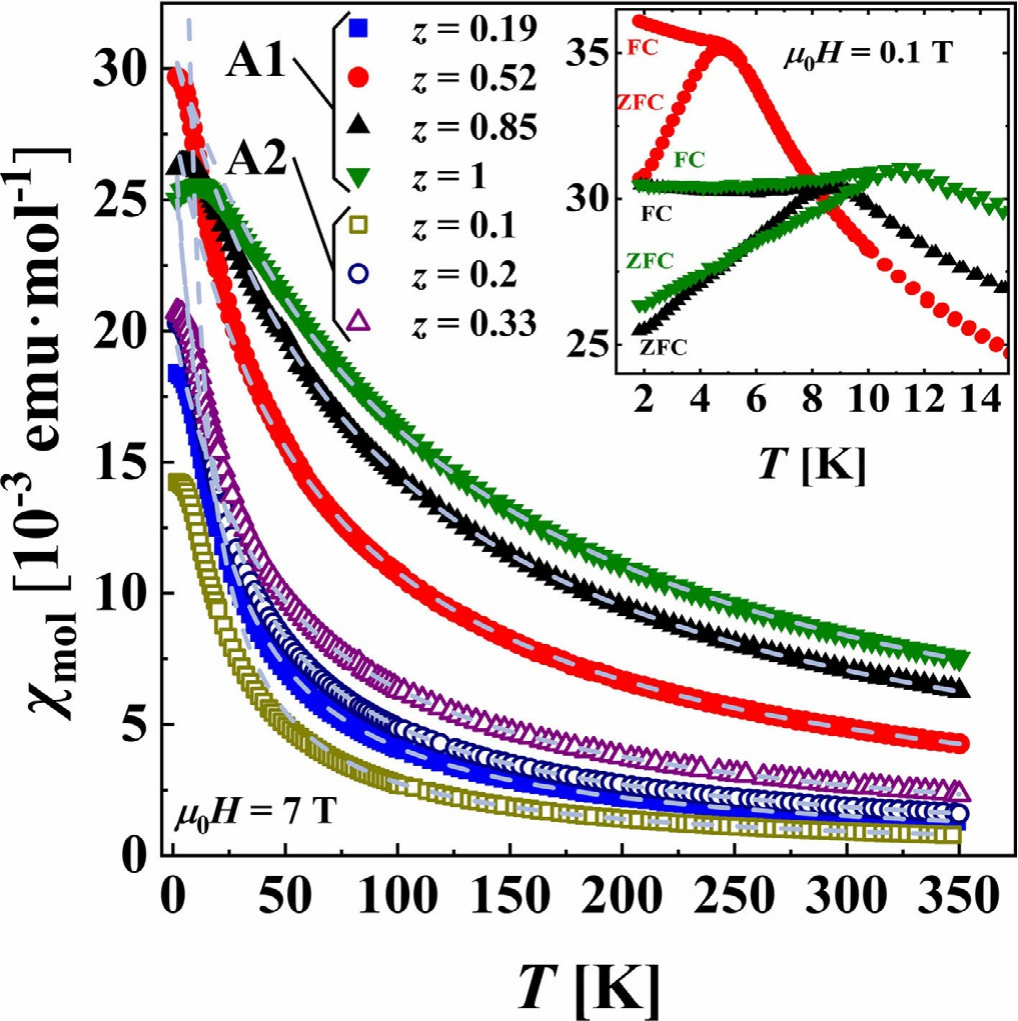
Molar magnetic susceptibility (*χ*_mol_) of (In_0.67+0.33*y*_)[In_2−*z*_Fe_*z*_]S_4_ (A1) and (In_0.67+*y*_)[In_2−*z*_Fe_*z*_]S_4_ (A2) samples (*y*=*z*) at an external field (*μ*_0_ *H*) of 7 T. Dashed lines indicate Curie–Weiss‐law fits for the temperature (*T*) range 100–350 K. Inset: field cooling (FC) and zero‐field cooling (ZFC) measurements at 0.1 T for the samples with antiferromagnetic (AF) ordering.

The magnetic susceptibilities of iron indium thiospinel are depicted in Figure 5. They increase for larger Fe‐content *z* in the sample (independently of the series), and above 100 K they can be fitted to the Curie–Weiss (CW) law. Effective magnetic moments (*μ*_eff_) and Weiss temperatures (*θ*_CW_), obtained from the fit, are collected in Table 4. The *μ*_eff_ varies in the range of 4.54–5.28 *μ*_B_, which is close to the theoretically calculated μtheoeff =4.90 *μ*_B_ for free Fe^2+^ ions. The Weiss temperatures are negative for all specimens reported here and decrease with increasing Fe content in the samples. Despite such clear indications for antiferromagnetic (AF) interactions, AF ordering is observed only for thiospinel with *z*=0.52, 0.85, and 1 [divergences of *χ*_mol_(*T*) between field‐cooling (FC) and zero‐field‐cooling (ZFC) modes, characteristic for AF transitions, are shown in the inset of Figure 5]. Taking into account that *θ*_CW_/*T*_N_ ratios vary within 10–13, one can expect AF ordering for the other compositions of iron indium thiospinel well below 1.8 K (beyond the temperature range accessible with our magnetometer). Our data for stoichiometric (In)[FeIn]S_4_ are in good agreement with earlier reports.^35^, ^37^, ^38^

### Thermal stability during thermoelectric measurements

According to differential scanning calorimetry (DSC) analysis, the obtained A1‐ and A2‐samples are definitely stable up to 873 K and reveal no thermal effects. Although heating and cooling curves of the thermal conductivity *κ*_tot_(*T*) above RT coincide with each other for all samples, this is not the case for the electrical resistivity *ρ*(*T*) and the Seebeck coefficient *α*(*T*). Small differences in *ρ*(*T*) and *α*(*T*) for (In_0.95_)[Fe_0.85_In_1.15_]S_4_ and (In)[FeIn]S_4_ become more pronounced when the vacancy concentration increases, in particular for the samples with *z*=0.1, 0.19, and 0.2 (Figure S7, Supporting Information). Values of *α*(*T*) and *ρ*(*T*) derived from the cooling curves are reproducible in the following measurement cycles. Such behavior can probably be rationalized with changes in values and temperature dependence of the charge carrier mobility after HT measurement and consequently different scattering mechanisms (Figure S8, Supporting Information). We did not observe any decomposition or any significant change of the lattice parameters after experiments at ZEM‐3. As it was proposed previously,^20^, ^39^ the distribution of cations in the spinel structure depends on temperature. If such phenomena exist in In‐thiospinels as well, they would affect the electronic and thermal transport properties. To propose solid conclusions from our observations, a quantitative structure analysis (Mössbauer or vibrational spectroscopy) should be carried out at elevated temperatures. In the following text, thermoelectric properties from the 1^st^ heating measurement are discussed.

### Electronic transport properties

**Figure 6:**
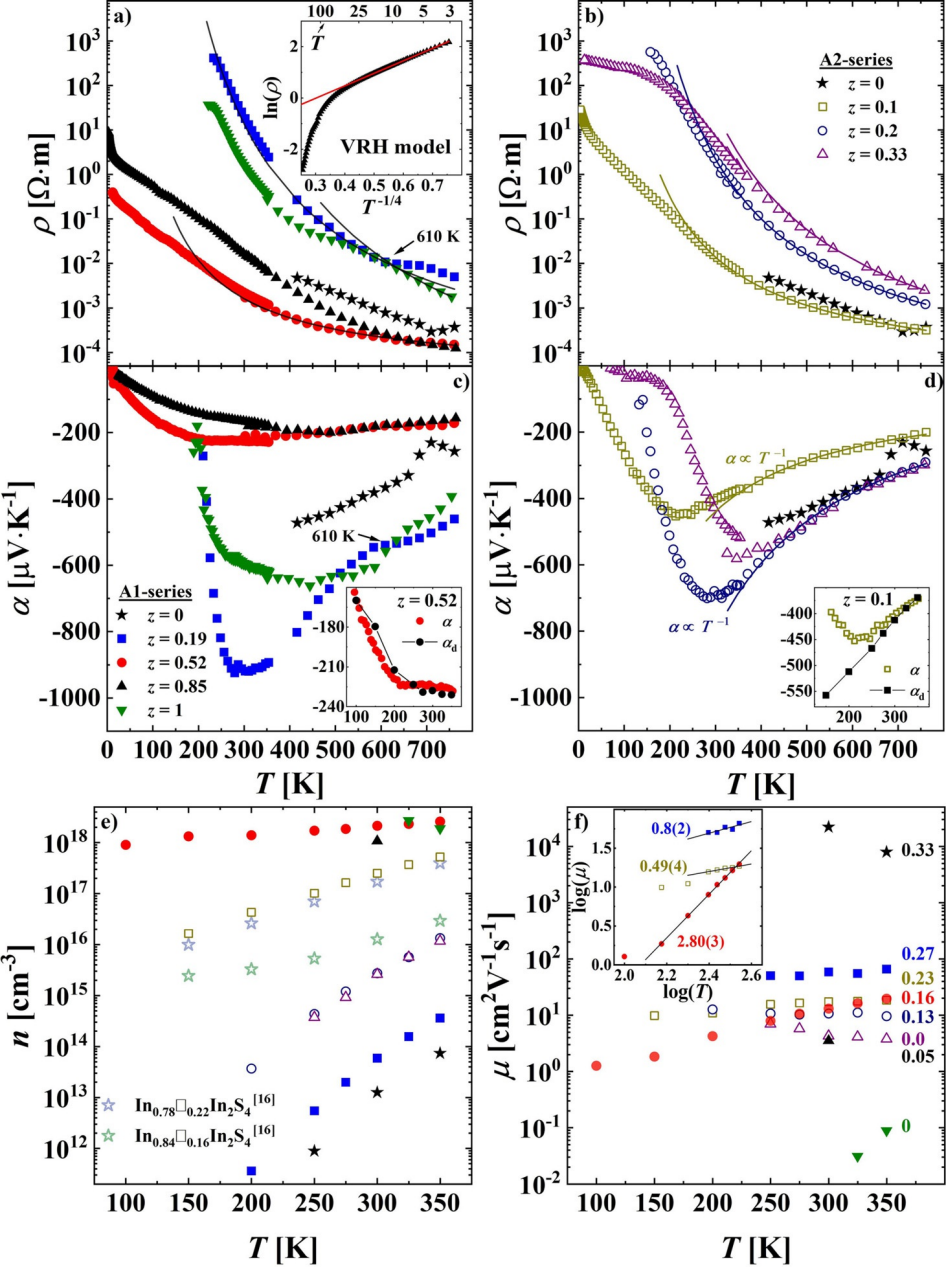
Electronic transport properties of the (In_0.67+0.33*y*_)[In_2−*z*_Fe_*z*_]S_4_ (A1) and (In_0.67+*y*_)[In_2−*z*_Fe_*z*_]S_4_ (A2) samples (*y*=*z*). The color code is the same for all figures. a, b) Electrical resistivity. *ρ*(*T*)=*ρ*_0_×exp[*E*_a_/(2*kT*)] dependences are drawn with solid lines. Due to the smaller numerical error, the Arrhenius relation was first fitted to electrical conductivity data and then recalculated for *ρ*. Inset in a): variable‐range hopping (VRH) model for (In_0.95_)[Fe_0.85_In_1.15_]S_4_ fitted to the equation: ln (*ρ*)=−1.48(1)+4.86(2)×*T* ^−1/4^. c, d) Seebeck coefficient. Fitted *α*∝*T* ^−1^ dependences: −55(4)−11.5(2)×10^4^×*T* ^−1^ for (In_0.77_)[Fe_0.1_In_1.9_]S_4_ and −36(6)−25.2(4)×10^4^×*T* ^−1^ for (In_0.87_)[Fe_0.2_In_1.8_]S_4_. Insets: comparison of the experimental Seebeck coefficient *α* and the electron‐diffusion contribution *α*_d_ calculated from the equation according to Herring^40^ for the effective electron mass m*=0.08 m_0_ (better agreement than for m*=0.09 m_0_ calculated from SPB model; see below). e) Charge carrier concentration. (f) Charge carrier mobility. Numbers indicate the nominal vacancy concentration per formula unit. Inset: *μ*(*T*) dependence in a double logarithmic plot.

The electrical resistivity and the Seebeck coefficient were measured in the low‐temperature (LT) (<350 K) and HT (>300 K) regime with PPMS and ZEM‐3 devices, respectively. Both data sets perfectly coincide with each other, confirming the homogeneity of the pellets after SPS fabrication (Figure 6 a–d).

The A1‐ and A2‐samples reveal typical semiconducting properties: 1) electrical resistivity between 10^−4^ and 10^3^ Ω m and 2) a monotonous decrease of *ρ*(*T*) with increasing temperature (Figure 6 a, b). The region of intrinsic conduction {that is, where *ρ*(*T*)∝exp[*E*_a_/(2*kT*)], where *k* is Boltzmann constant} was observed above 300 K (solid lines in Figure 6 a, b), with calculated activation energies *E*_a_ given in Table 5. For (In_0.84_)[Fe_0.52_In_1.48_]S_4_ and all A2‐samples, single *E*_a_ values describe the whole HT region, whereas for other specimens the *ρ*(*T*) trend is more complex (*E*_a_ values for the highest temperature range are presented in Table 5). Although the sample with *z*=0.33 contains two impurity phases, it shows *ρ*(*T*) similar to that of (In_0.87_)[Fe_0.2_In_1.8_]S_4_. Consequently, a plateau between 3 and 200 K can be associated with a saturation range of impurity conduction of the iron indium thiospinel within the A2‐series (Figure 6 b). The steep increase in *ρ* below 25 K for the sample with *z*=0.85 follows variable‐range hopping (VRH) behavior with ln(*ρ*)=ln(*ρ*_0_)+(*T*_0_/*T*)^−1/4^ (inset in Figure 6 a).

**Table 5:** Activation energy *E*_a_ (within the given temperature range), charge carrier concentration *n*, charge carrier mobility *μ*, and effective electron mass m* at 300 K.

Series	*z*	*E*_a_ [eV]	*α* [μV K^−1^]	*n* [cm^−3^]	*μ* [cm^2^ V^−1^ s^−1^]	m*/*m*_0_
A1	0	^[a]^	−470 (415 K)	1.3×10^13^	2.2×10^4^	–
	0.19	0.73 (415–610 K)	−919	5.9×10^13^	58.7	–
	0.52	0.21 (300–760 K)	−210	2.1×10^18^	13.1	0.09
	0.85	0.37 (587–764 K)	−167	1.1×10^18^	3.5	0.04
	1	1.03 (634–755 K)	−593	(≈4×10^18^)^[b]^	(≈10^−2^)^[b]^	–
						
A2	0.1	0.32 (295–760 K)	−402	2.5×10^17^	17.4	–
	0.2	0.64 (370–760 K)	−683	2.7×10^15^	10.7	–
	0.33	0.79 (509–757 K)	−438	2.6×10^15^	4.3	–

[a] Electrical resistivity of In_0.67_□_0.33_In_2.67_S_4_ does not follow a simple exponential trend in the studied temperature range. [b] Estimated values.

In contrast to a previous report on the energy gap *E*_g_ in a single crystalline iron indium thiospinel,^24^ we did not recognize any clear dependence of *ρ*(*T*) or *E*_a_ versus the Fe content (or the vacancy concentration) in the A1‐series. The lowest resistivity is observed for (In_0.84_)[Fe_0.52_In_1.48_]S_4_ with intermediate Fe content. For the A2‐samples, *E*_a_ and *ρ*(*T*) increase monotonously with decreasing vacancy concentration. The fact that *E*_a_ is much smaller than *E*_g_ (e.g., by a factor of 2 for (In)[FeIn]S_4_)^24^ suggests the presence of an impurity band within the energy gap of this thiospinel.

For all samples, the Seebeck coefficient *α*(*T*), as well as the Hall coefficient *R*_H_ (Figure S8 a, Supporting Information), are negative in the whole measured temperature range. This indicates electrons are the main charge carriers (Figure 6 c, d). Large values |*α*(*T*)|>200 μV K^−1^ above 100 K correspond to high *ρ*(*T*), in agreement with the proportionality of these two parameters.^41^ Again, no correlation between *α*(*T*) and the Fe content (or the vacancy concentration) was found. The values of the Seebeck coefficient approaches 0 at low temperature, and *α*(*T*) dependences show a minimum for most compositions (weak *T* dependence for samples with *z*=0.52 and 0.85), similar to binary In_0.78_□_0.22_In_2_S_4_.^16^

**Figure 7:**
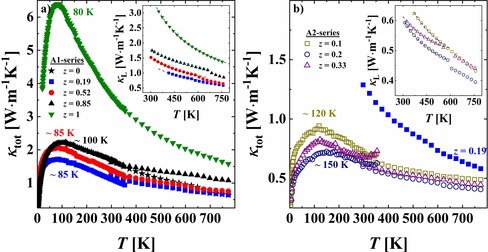
Total thermal conductivity *κ*_tot_ for a) the A1‐series and b) the A2‐series (sample with *z*=0.19 shown for comparison). Upturns around 300 K in the LT sets of *κ*_tot_ are caused by an increased radiation contribution during the measurement. Insets in a) and b): lattice contribution to the thermal conductivity. Dashed line indicates *κ*_L_∝*T* ^−1^ trend.

The most pronounced minima are observed for the samples with *z*=0.1, 0.19, 0.2, and 0.33 at 220, 300, 290, and 390 K, respectively (Figure 6 c, d). Peak temperatures increase with the Fe content, and the A2‐samples show *α*∝*T* ^−1^ behavior above the peak. Given that these anomalies are not observed for *χ*(*T*), *ρ*(*T*), *κ*_tot_(*T*), or the heat capacity *C*_p_(*T*) dependences (Figure 5, Figure 6, Figure 7 a), one may exclude the possibility of any structural or magnetic transition. There is also no variation of *α*(*T*) with magnetic field (Figure S9 a, Supporting Information), which indicates a non‐electronic origin of this effect.^42^ Another possible explanation may be a phonon‐drag contribution *α*_g_ to the Seebeck coefficient *α*=*α*_d_+*α*_g_, in which *α*_d_ is a conventional electron‐diffusion component. To verify the possibility of such a scenario, the diffusion contributions were calculated for (In_0.77_)[Fe_0.1_In_1.9_]S_4_ [a sample from the A2‐series with a minimum in *α*(*T*) at 220 K] and (In_0.84_)[Fe_0.52_In_1.48_]S_4_ [a sample from the A1 series without an anomaly in *α*(*T*)] specimens, by using an equation derived by Herring^40^ [Eq. (1)] (in the range of 100–350 K):

(1) $$\alpha {}_{d}=-\;86.2\times [\ln\;\left(4.70\times 10{}^{15}/n\right)+1.5\times \ln\;\left(m{}^{*}\right)+2.5+r+1.5\times \ln\;(T)]$$

in which *n*(*T*) is the charge carrier concentration (deduced from Hall effect measurements), *r*=1.5 is a scattering factor derived from the charge carrier mobility *μ*(*T*) data, and m*=0.08 is the effective electron mass (see discussion below). As one can see from the inset in Figure 6 c, the calculated *α*_d_(*T*) is almost coinciding with the experimental *α*(*T*) (i.e., *α*_d_ is the dominating component) for (In_0.84_)[Fe_0.52_In_1.48_]S_4_ thiospinel. On the other hand, *α*_d_(*T*) is resembling experimental values for (In_0.77_)[Fe_0.1_In_1.9_]S_4_ only above 250 K (inset in Figure 6 d). Such a behavior may be a hint toward a phonon‐drag effect in samples with minima in the *α*(*T*) dependence.^42^ To prove this, a precise study of carrier concentration, coupled with electronic calculations of transport properties, is required.

The charge carrier concentration *n*(*T*) increases with temperature for all samples, as expected for semiconductors (Figure 6 e). For the A1‐series, *n* gradually grows with the Fe content from 5.9×10^13^ cm^−3^ for *z*=0.19 up to 2.1×10^18^ cm^−3^ for *z*=0.52 at RT, and then the carrier concentration seems to be saturated at the level of approximately 10^18^ cm^−3^ for samples with *z*=0.85 and 1 (Table 5). On the other hand, *n*(*T*) becomes smaller for samples with larger Fe content (i.e., lower vacancy concentration) within the A2‐series (Figure 6 e). Interestingly, *n*(*T*) for the cubic binary In_0.84_□_0.16_In_2_S_4_ thiospinel is smaller than that of In_0.78_□_0.22_In_2_S_4_, which would hint toward the same trend observed for the Fe‐containing A2‐series [reduction of *n*(*T*) with decreasing vacancies concentration; Figure 6 e]. This effect can be attributed to the same structural peculiarities of both series, which are 1) charge imbalance with incorporation of additional In/Fe and 2) tetrahedral voids occupied exclusively by In atoms.^16^

We also present the *n*(*T*) for tetragonal binary β‐In_0.67_□_0.33_In_2_S_4_ in Figure 6 e. If it would be considered as a sample with *z*=0, its *n*(*T*) would also follow the above‐discussed trend for the A1‐series and disagree with this for the A2‐series and the In_1−*x*_□_*x*_In_2_S_4_ thiospinel (*x*<0.33). Such a behavior of *n*(*T*) of β‐In_0.67_□_0.33_In_2_S_4_ can be explained by the fact that this sulfide is charge‐balanced (as is the A1‐series).

A clear reduction of the charge carrier mobility *μ* for lower vacancy concentration (see numbers in Figure 6 f) was observed for all samples (independent of the series) above 275 K, similar to the cubic In_1−*x*_□_*x*_In_2_S_4_ compound.^16^ It seems that *μ* can be considered as an indicator of vacancy concentration in the studied thiospinel. Interestingly, *μ*(350 K) reaches a value of approximately 10^−1^ cm^2^ V^−1^ s^−1^ for (In)[FeIn]S_4_, which is much lower than values observed for the other studied specimens. Such a low mobility may suggest small‐polaron hopping (SPH) mechanism of conduction,^43^, ^44^ which is frequently reported for spinel compounds.^45^, ^46^, ^47^, ^48^, ^49^ However, analysis of the *α*(*T*) dependence for (In)[FeIn]S_4_ provides ambiguous results (see Figure S9 and discussion in Supporting Information), and thus more detailed studies of *μ*(*T*) in this thiospinel are required.

Both *μ* (Figure 6 f) and log(*μ*) (inset in Figure 6 f) for the ternary A1‐ and A2‐series increase with temperature and log(*T*), respectively (with the only exception being the three‐phase (In)[Fe_0.33_In_1.67_]S_4_). The same behavior was observed for the cubic binary In‐S thiospinel.^16^ In relaxation time approximation (*τ*=*τ*_0_×*E* ^*r*^×*T* ^−*s*^) [assuming effective electron mass m*(*E*,*T*)=constant], the carrier mobility *μ* is proportional to *T* ^*r*−*s*^, in which *r* is the scattering parameter [i.e., *r*=−0.5 or 1.5 for acoustic phonon scattering (APS) or ionized impurity scattering (IIS), respectively], and *s* is the number of phonons taking part in the scattering (typically *s*=0 and 1 for IIS and APS, respectively).^50^, ^51^, ^52^ Consequently, (*r*−*s*)=−0.5 and 1.5 are expected for APS and IIS, respectively. Linear dependences log(*μ*) versus log(*T*) are observed for samples with the Fe‐content *z*=0.1, 0.19, and 0.52, for which the slopes (*r*−*s*)=0.49(4), 0.8(2), and 2.80(3), respectively, (inset in Figure 6 f) were obtained. This finding would indicate the IIS mechanism to be the dominating one among other possible mechanisms (e.g., APS), given that the values of (*r*−*s*) are not exactly 1.5.

The *α*(*T*) dependence without any anomalies (i.e., maxima/minima) for the thiospinel with the Fe‐content *z*=0.52 and *z*=0.85 (Figure 6 c) allowed us to estimate the effective electron mass m* for these compositions at 300 K (Table 5) by using a single parabolic band (SPB) model (for more details, see Supporting Information). The obtained values of 0.09m_0_ (*z*=0.52) and 0.04m_0_ (*z*=0.85) are similar to those for binary In‐based semiconductors^53^, ^54^ and much smaller than m* reported for the *TT′*_2_ *Ch*_4_ (*T*=Cu, Zn, Cd, Sn, Hg; *T′*=Al, Ga, In, Ti, Co, Cd; *Ch*=O, S) spinels.^55^, ^56^, ^57^, ^58^

### Thermal transport properties and thermoelectric figure of merit

The total thermal conductivity *κ*_tot_ for (In)[FeIn]S_4_ (the sample from the A1‐series without vacancies) presents a *T* dependence typical for ordered crystalline materials [that is, a well‐resolved maximum at 80 K, which separates boundary‐scattering (<80 K) and Umklapp‐scattering (>80 K) regions], as well as a relatively high *κ*_tot_=3.6 W m^−1^ K^−1^ at 300 K (Figure 7 a). Interestingly, after a small reduction of the Fe content (i.e., increasing vacancy concentration) for (In_0.95_)[Fe_0.85_In_1.15_)S_4_, the *κ*_tot_(*T*) trend becomes more similar to dependences observed for disordered or amorphous compounds^59^, ^60^ (i.e., the width of the peak increases), and *κ*_tot_ decreases to 1.8 W m^−1^ K^−1^ at 300 K. The presence of vacancies clearly strengthens phonon scattering and promotes low *κ*_tot_ in this series. With further lowering of the Fe content, *κ*_tot_ still decreases but less pronouncedly. The total thermal conductivity of (In_0.84_)[Fe_0.52_In_1.48_]S_4_ is very close to the values for ordered β‐In_0.67_□_0.33_In_2_S_4_.

The thermal conductivity of iron indium thiospinel from the A2‐series [*κ*_tot_(*T*)<0.7 W m^−1^ K^−1^ above RT; Figure 7 b] is remarkably lower than that of the A1‐series (charge balance) and is rather comparable to those for cubic In_1−*x*_□_*x*_In_2_S_4_ compounds^16^ (charge imbalance). It decreases slightly for larger Fe content. Obviously, the charge imbalance plays a crucial role in a reduction of the thermal conductivity and leads to the disordered character of *κ*_tot_(*T*) for the A2‐samples.

The lattice part *κ*_L_ dominates *κ*_tot_ for all samples, and its contribution decreases from 99 % for (In_0.73_)[Fe_0.19_In_1.81_]S_4_ to 82 % for (In_0.84_)[Fe_0.52_In_1.48_]S_4_, in agreement with the measured resistivity (Figure 6 a, b and insets in Figure 7 a, b). Umklapp‐type behavior *κ*_L_∝*T* ^−1^ is visible for all samples above RT (insets in Figure 7 a, b). For all specimens with vacancies (except for the sample with *z*=1), there is a weak anomaly in *κ*_L_(*T*) between 525 and 625 K and its temperature increases with the Fe content. The origin of the anomaly is not clear now and would require some additional studies.

**Figure 8:**
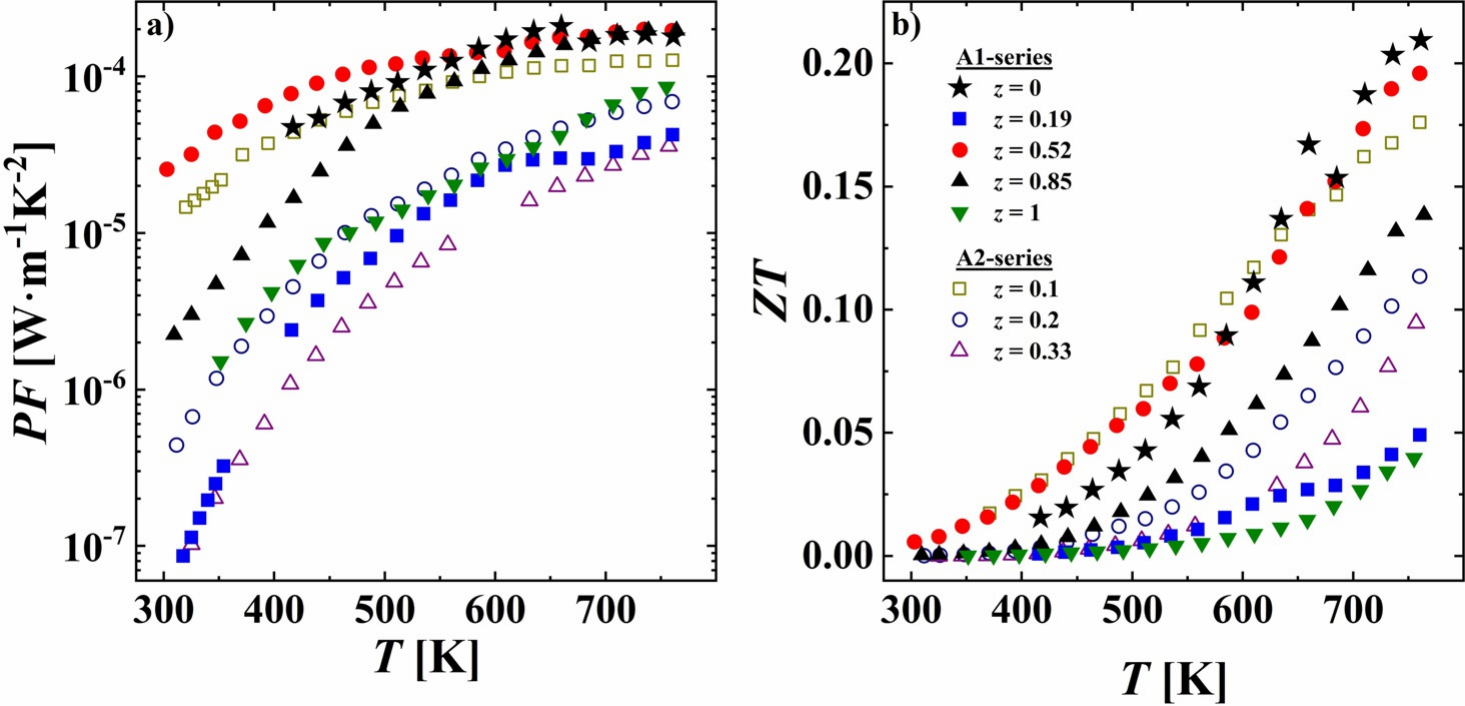
a) Power factor *PF* and b) thermoelectric figure of merit *ZT* as a function of temperature.

The power factor *PF* versus Fe content for iron indium thiospinel follows the trend of the electrical resistivity, and thus the highest values were obtained for (In_0.84_)[Fe_0.52_In_1.48_]S_4_ and (In_0.77_)[Fe_0.1_In_1.9_]S_4_ (Figure 8 a). Consequently, these samples exhibit the largest thermoelectric figure of merit *ZT* in the whole studied temperature range (Figure 8 b). However, there is no improvement of *ZT* in comparison to that of In_0.67_□_0.33_In_2_S_4_. As expected, Fe incorporation enhanced the charge carrier concentration of indium thiospinel. However, such an enhancement does not sufficiently reduce the electrical resistivity and is accompanied with a remarkable decrease (by approximately 50 % at 400 K) of the Seebeck coefficient. Combination of these two factors does not allow improvement of the TE efficiency of In thiospinel.

## Conclusion

Four series of Fe‐In‐S samples, described by formulas (In_0.67+0.33*y*_□_0.33−0.33*y*_)_tetr_[In_2−*z*_Fe_*z*_]_oct_S_4_ (*z*=*y*=0, 0.19, 0.52, 0.85, 1; A1, charge balance), (In_0.67+*y*_□_0.33−*y*_)_tetr_[In_2−*z*_Fe_*z*_]_oct_S_4_ (*z*=*y*=0.1, 0.2, 0.33; A2, charge imbalance), (In)_tetr_[In_2−*z*_Fe_*z*_]_oct_S_4_ (*z*=0.53, 0.73, 0.95, 0.97; A3), and Fe_0.345_In_2.552_S_4_ (A4), were synthesized by long‐term annealing at 873 K, followed by quenching in liquid nitrogen. Based on powder X‐ray diffraction (PXRD) phase analysis and wavelength‐dispersive X‐ray spectroscopy (WDXS) investigations, the homogeneity range of iron indium thiospinel at 873 K was established.

Comparison of the intensities of experimental and theoretically calculated (assuming Fe atoms occupy different crystallographic positions) PXRD reflections, crystal structure refinements, as well as ^57^Fe Mössbauer spectroscopy for the A1‐ and A2‐samples revealed Fe atoms occupying the octahedral voids only in the studied thiospinel.

Both series show an unusual decrease of the unit cell parameter with increasing Fe content (i.e., lowering of the vacancy concentration). We recognized the same trend in the UCP behavior for indium thiospinels, in which *r* ${}_{M{}^{1+}}$ /*r* ${}_{M{}^{2+}}$ <*r* ${}_{\mathrm{In}{}^{3+}}$ (*M*=Co, Ni, Cu). In the cases for which *r* ${}_{M{}^{1+}}$ /*r* ${}_{M{}^{2+}}$ >*r* ${}_{\mathrm{In}{}^{3+}}$ (*M*=Na, Ag, Cd), UCP increases for larger *M* content. The steeper reduction of UCP for the A2‐series in comparison with that of the A1‐series is ascribed to an additional electrostatic attraction of In^3+^ and S^2−^ ions within the tetrahedra, caused by charge imbalance.

Magnetic susceptibility measurements revealed 1) effective magnetic moments to be close to the theoretically calculated one for free Fe^2+^ ions, 2) an enhancement of antiferromagnetic (AF) interactions with increasing Fe content (independent of the studied series), and 3) AF ordering for samples with *z*=0.52, 0.85, and 1 (transition Néel temperature *T_N_* increases with increasing *z*).

The studied thiospinel shows a semiconductor‐like temperature dependence of the electrical resistivity. Activation energies deduced from these measurements do not follow any trend as a function of the Fe content in the A1‐series and they increase for larger Fe content for the A2‐series.

The Seebeck coefficient *α*(*T*) is negative in the whole measured temperature range for all samples, which indicates electrons to be the main charge carriers. This parameter also shows no correlation with the Fe content in the compound. The observed minima in *α*(*T*) cannot be described with a diffusion contribution *α*_d_(*T*) only and suggest a phonon‐drag effect.

The charge carrier concentration *n*(*T*) gradually increases and decreases with the Fe content for the A1‐ and A2‐series, respectively. The A2‐series shows the same trend as that observed for the binary In_1−*x*_□_*x*_In_2_S_4_ thiospinel.

The charge carrier mobility *μ* decreases with the vacancy concentration for all samples (independent from the series). The analysis of the *μ*(*T*) dependences indicated ionized impurity scattering to be a dominating scattering mechanism of charge carriers in the studied thiospinels.

The thermal conductivity *κ*_tot_(*T*) decreases for lower Fe content for the A1‐series and is nearly the same within the A2‐series. Such a dependence can be explained by the introduction of additional structural disorder and thus enhanced phonon scattering. In the studied thiospinel, *κ*_tot_(*T*) is dominated by the lattice contribution. The A2‐samples reveal the lowest values of *κ*_tot_(*T*)<0.7 W m^−1^ K^−1^ above RT.

The best thermoelectric (TE) performance (*ZT*≈0.2 at 750 K) is observed for the (In_0.84_)[Fe_0.52_In_1.48_]S_4_ and (In_0.77_)[Fe_0.1_In_1.9_]S_4_ specimens, which is comparable with values recently reported for the binary In_0.67_□_0.33_In_2_S_4_ thiospinel. An enhancement of charge carrier concentration is not sufficient to improve the TE efficiency of such a compound. It has to be additionally accompanied with the simultaneous optimization of the electron effective mass and charge carrier mobility through electronic structure modification (e.g., band sharpening, band convergence). We propose this could be realized by substitution of sulfur by isoelectronic elements, that is, Se or Te.

## Experimental Section

### Synthesis and sample preparation

Thirteen samples from the ternary Fe‐In‐S system were synthesized (Figure 1, Table 1). Powders of elemental iron (99.998 %, −22 mesh, Alfa Aesar), indium (99.99 %, −325 mesh, Alfa Aesar), and sulfur (99.5 %, −325 mesh, Alfa Aesar) were mixed in appropriate ratios (approximately 2–3 g in total), cold‐pressed, and sealed in quartz tubes under vacuum (<2×10^−4^ mbar). To minimize the influence of oxygen and moisture, all manipulations were done in an argon‐filled glovebox [MBraun, *p*(O_2_/H_2_O) <0.1 ppm]. Annealing of the Fe‐In‐S samples at 873 K for 168 h was sufficient to complete a chemical reaction for specimens with the nominal Fe content ≤7.6 at. % only (samples with *z*=0.1, 0.19, 0.2, 0.33, and 0.52; Table 1). The other samples were first reacted at 1073 K for 72 or 336 h and then equilibrated at 873 K for 168 h. Each annealing step was followed by quenching in liquid nitrogen or iced water.

For the measurement of thermoelectric properties, selected samples were manually ground in an agate mortar and compacted using a spark plasma sintering apparatus placed in an argon‐filled glovebox (515ET Sinter Lab, Fuji Electronic Industrial Co. Ltd., 923 K, 10 min, 75 MPa, graphite dies).^61^ Obtained cylindrical pellets (*ϕ*=10 mm, *h*=1–2 mm) were manually polished, graphite‐coated, and used for the thermal diffusivity measurements. Further, the pellets were cut into bars (approximately 8×2×2 mm^3^) that were used for the other transport measurements.

### X‐ray diffraction

All samples were characterized by powder X‐ray diffraction by using a Huber G670 Guinier camera [CoK_α1_ radiation, *λ*=1.788996(1) Å, 2*θ*_max_=100°, Δ2*θ*=0.005°, LaB_6_ as internal standard (NIST 660a, *a*=4.156916(1) Å)]. Phase analysis and simulation of theoretical PXRD patterns were done with the WinXPow software.^62^ The WinCSD package^63^ was used to refine 1) lattice parameters using least‐square method and 2) the crystal structure by Rietveld analysis.

### Differential scanning calorimetry

The differential scanning calorimetry of the Fe‐In‐S samples after SPS treatment was performed by using a DSC 404C Pegasus (Netzsch, temperature range 298–873 K, heating/cooling rate: 10 K min^−1^, Al_2_O_3_ crucibles, Ar flow). No thermal effects were observed up to 873 K.

### Metallography, scanning electron microscopy, and wavelength‐dispersive X‐ray spectroscopy

Samples for the metallographic characterization (A1‐ and A2‐series, Table 1) were embedded in a conductive resin and polished with a SiC abrasive paper and with a diamond polishing solution mixed with a water‐free lubricant (semi‐automatic system, EcoMet 250 pro, Buehler). Microstructural analysis was carried out by optical microscopy (Axioplan 2, Carl Zeiss) and scanning electron microscopy (SEM, Jeol 7800F, Field emission FEG) with an energy‐dispersive X‐ray spectrometer (Quantax 400, Bruker, e^−^ Flash^HR^, silicon drift detector). As a result of notable differences in the atomic masses between S and both Fe and In, as well as the possibility of S evaporation from the surface in the electron beam, EDXS yields an unreliable S concentration. Thus, the more accurate wavelength‐dispersive X‐ray spectroscopy method was applied (Cameca SX 100, PeakSight ver. 5.21, Fe K_α_, In L_α_, S K_α_, reference materials: single crystals of FeS_2_ and In_2_S_3_). Determined chemical compositions are presented in Table 1.

### Mössbauer spectroscopy

A ^57^Co/Rh source was used for the Mössbauer spectroscopic measurements. The measurements were conducted in transmission geometry in a continuous flow cryostat system (Janis Research Co LLC) at 6 K, while the source was kept at room temperature. The samples were placed in poly(methyl methacrylate) (PMMA) containers with an optimized thickness as described before.^64^ The WinNormos routine^65^ was used to fit the spectra. The data collection times were 4 days for the (In_0.77_)[Fe_0.1_In_1.9_]S_4,_ 3 days for the (In_0.87_)[Fe_0.2_In_1.8_]S_4,_ 5 days for the (In)[Fe_0.33_In_1.67_]S_4_, and 2 days for the (In_0.84_)[Fe_0.52_In_1.48_]S_4_ samples.

### Magnetic and thermoelectric measurements

Low‐temperature (1.8–400 K) magnetic susceptibility *χ*_mol_ was measured with a superconducting quantum interference device magnetometer (MPMS XL‐7, Quantum Design) using external magnetic fields of 7, 3.5, 0.1, and 0.002 T. An effective magnetic moment *μ*_eff_=(7.977 *C* *m* ^−1^)^0.5^ of Fe was determined by fitting *χ*_mol_(*T*)=*C*×(*T*−*θ*_CW_)^−1^, in which *C*=Curie constant, *m*=number of Fe atoms per formula unit, and *θ*_CW_=paramagnetic Weiss temperature in Kelvin.

LT (3–350 K) TE properties [Seebeck coefficient *α*(*T*), electrical resistivity *ρ*(*T*), and thermal conductivity *κ*_tot_(*T*)] as well as the Hall coefficient *R*_H_(*T*) were measured with a PPMS (Quantum Design, Thermal Transport‐ and Resistivity‐options, respectively). The charge carrier concentration was calculated from *n*=(*e*×*R*_H_)^−1^ and the electron mobility from *μ*=(*ρ*×*n*×*e*)^−1^ (*e*=1.602×10^−19^ C).

High‐temperature (300–770 K) *ρ*(*T*) and *α*(*T*) were determined simultaneously using direct current (DC) four‐point and differential methods, respectively (ZEM‐3, Ulvac‐Riko).

HT thermal conductivity *κ*_tot_ was calculated from the relation *κ*_tot_=*a*_d_×*d*×*c*_p_. Thermal diffusivity *a*_d_ was measured by using laser flash analysis (LFA 457 MicroFlash Netzsch, HgCdTe detector, He flow). Sample density *d* was determined by using an immersion technique with ethanol as medium (*d*>98 % of theoretical density for all samples). Specific heat capacity *c*_p_ was measured by using a DSC 8500 (PerkinElmer, 298–773 K, 20 K min^−1^, Al_2_O_3_ crucibles, Ar flow, sapphire reference material). The Wiedemann–Franz law (*κ*_el_=*L*×*ρ* ^−1^×*T*, in which *L*=2.44×10^−8^ V^2^ K^−2^ is the Lorenz number) was used to decompose *κ*_tot_ into the electronic *κ*_el_ and the lattice (*κ*_L_=*κ*_tot_−*κ*_el_) contributions.

To estimate the TE efficiency of the studied materials, the power factor *PF* and the thermoelectric figure of merit *ZT* were calculated as: *PF*=*α* ^2^×*ρ* ^−1^ and *ZT*=*α* ^2^×(*ρ*×*κ*_tot_)^−1^×*T*. As a result of technical limitations, *a*_d_ (and consequently *κ*_tot_) at HT was measured parallel, whereas all LT properties as well as *ρ* and *α* at HT were measured perpendicular to the direction of applied pressure during the SPS process. Nevertheless, overlapping of *κ*_tot_(*T*) LT and HT values between 300–350 K indicate negligible anisotropy of *κ*_tot_ in the studied cubic thiospinel. Here we also assumed no anisotropy in the electronic transport properties. According to the manufacturer's data, measurement errors *s*(*κ*_tot_), *s*(*α*), *s*(*ρ*), and *s*(*ZT*) were estimated as 5, 10, 10, and 20 %, respectively.

## Conflict of interest

The authors declare no conflict of interest.

## Supporting information

As a service to our authors and readers, this journal provides supporting information supplied by the authors. Such materials are peer reviewed and may be re‐organized for online delivery, but are not copy‐edited or typeset. Technical support issues arising from supporting information (other than missing files) should be addressed to the authors.

Supplementary
