# Supplementary material for: Structural Peculiarities and Thermoelectric Study of Iron Indium Thiospinel
Source: Chemistry. 2020 Apr 6;26(23):5245–56. doi: 10.1002/chem.201905665 (PMC7216953; doi:10.1002/chem.201905665)
Supplement: Supplementary file 1 — Supplementary [file CHEM-26-5245-s001.pdf]

Chemistry–A European Journal

Supporting Information

Structural Peculiarities and Thermoelectric Study of Iron Indium Thiospinel

Paweł Wyżga,[a, b] Igor Veremchuk,[b] Matej Bobnar,[b] Primož Koželj,[b] Steffen Klenner,[c] Rainer Pöttgen,[c] Andreas Leithe-Jasper,[b] and Roman Gumeniuk*[a]

Table of content

Powder X-ray diffraction analysis 2

Optical and scanning electron microscopy 3

Comparison of unit cell parameters 5

Rietveld refinement analysis 6

Mössbauer spectroscopy 8

Electronic transport properties in the first heating-cooling cycle 9

Analysis of Seebeck coefficient (including small polaron hopping) 10

Single parabolic band model 11

References 12

Powder X-ray diffraction analysis

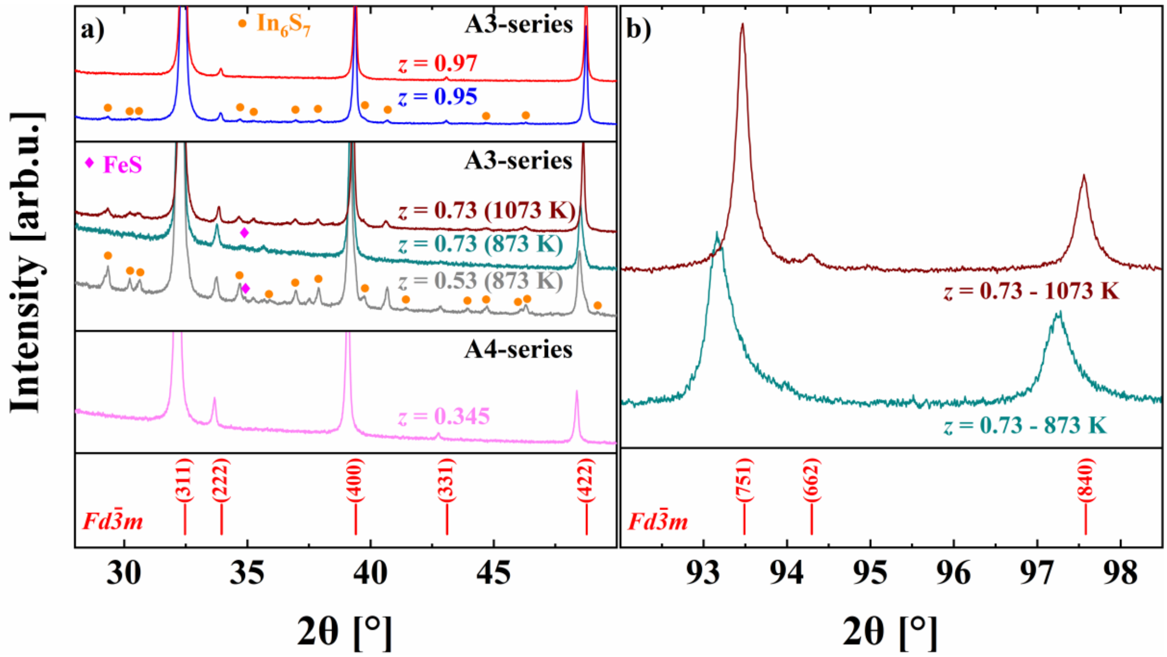

Figure S1. a) PXRD patterns of Fe-In-S samples after synthesis: A3- and A4-series, theoretical position of reflections for the structure with space group $Fd\bar{3}m$ (unit cell parameter as for (In)[FeIn]S_4, 4th panel). b) Patterns for the sample with z = 0.73 after synthesis at 873 K and annealing at 1073 K. Intensities in each pattern were normalized according to the reflection with the highest intensity.

The iron indium thiospinel within the A3-series was always accompanied with In_6S_7 and/or Fe-S phases (Table 1 in the main text, Figure S1a). After synthesis at 873 K, the samples $Fe_{0.53}In_{2.47}S_4$ and $Fe_{0.73}In_{2.27}S_4$ contained two spinel phases (an additional 'shoulder' appeared for all spinel-related reflections, Figure S1b). Further reaction occurs at higher temperature (Figure S1b) and a reduction in width of the X-ray reflections was observed.

Optical and scanning electron microscopy

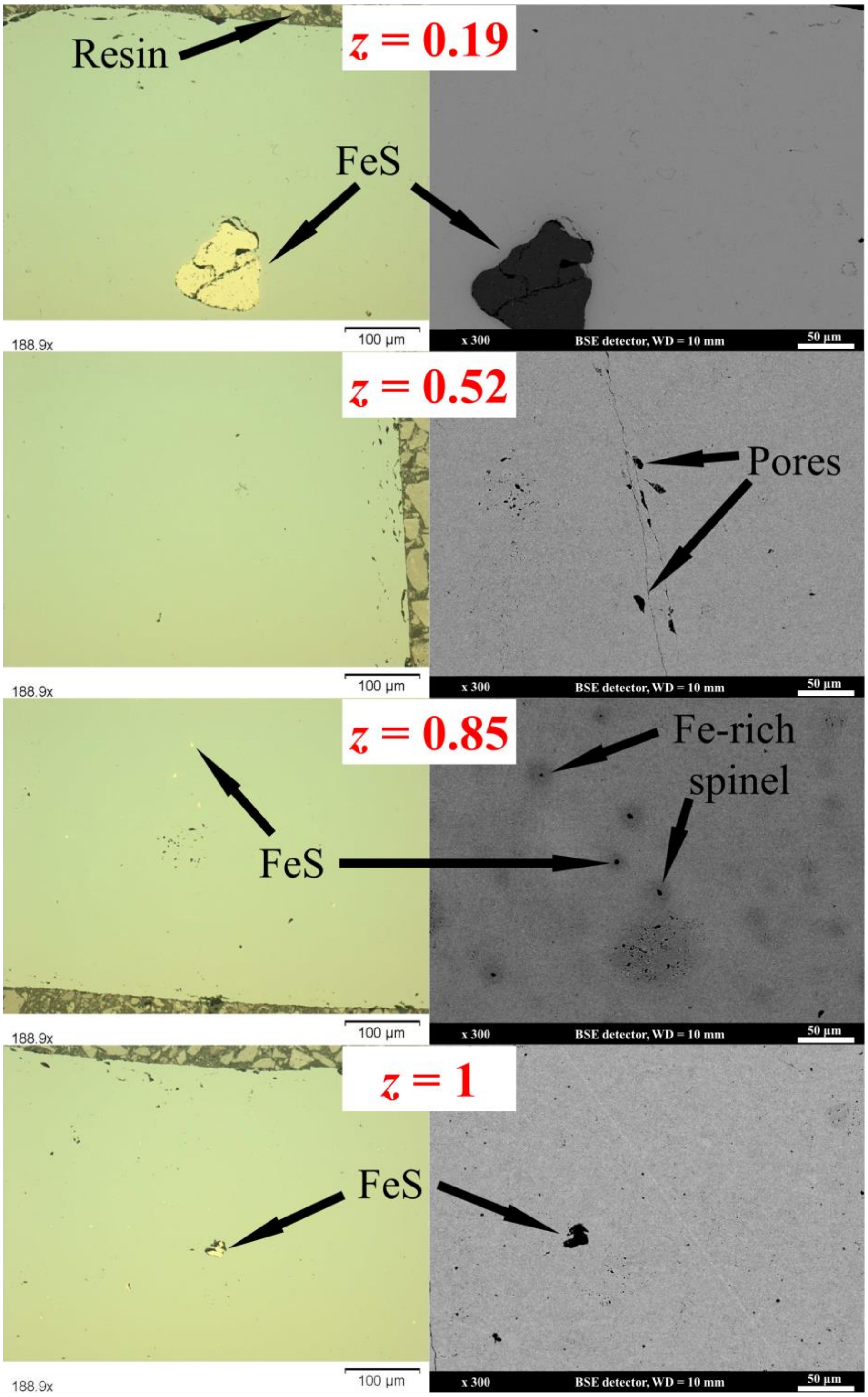

Figure S2. Optical bright-field (left panel) and backscatter electron (right panel) images for the A1-series.

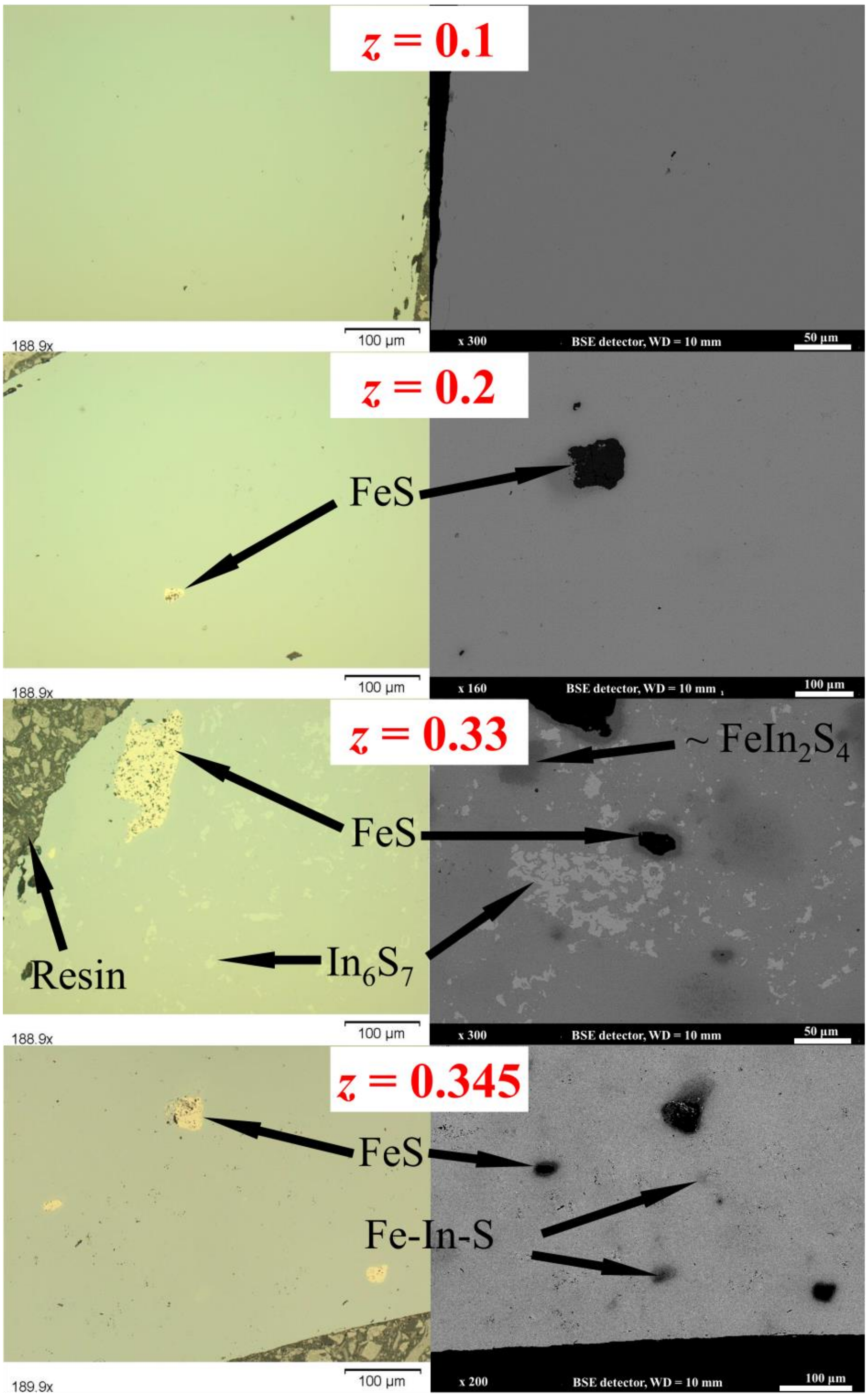

Figure S3. Optical bright-field (left panel) and backscatter electron (right panel) images for the A2- and A4-series.

Comparison of unit cell parameters

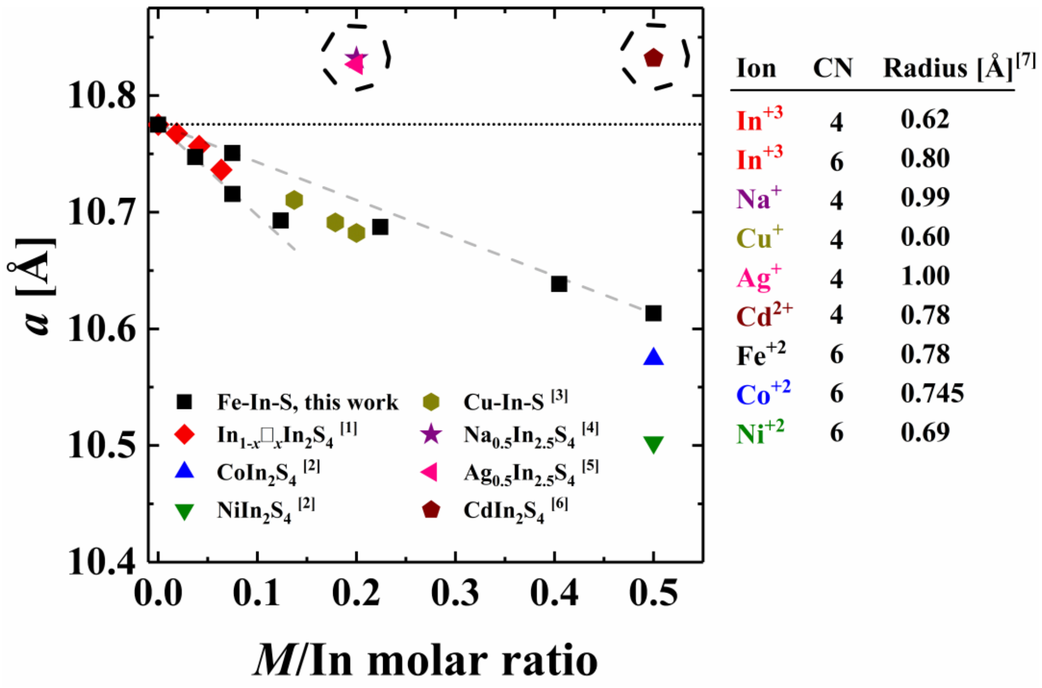

Figure S4. Unit cell parameters (UCPs) for $M_xIn_yS_4$ thiospinels (charge-balanced). M = Na, Cu, Ag, Cd, Fe, Co, Ni or In (excess of In in comparison with $In_{0.67}\square_{0.33}In_2S_4$ composition). Values larger than UCP of $In_{0.67}\square_{0.33}In_2S_4$ are highlighted by dashed-circles. Ionic radii according to Shannon.[7] CN - coordination number.

Rietveld refinement analysis

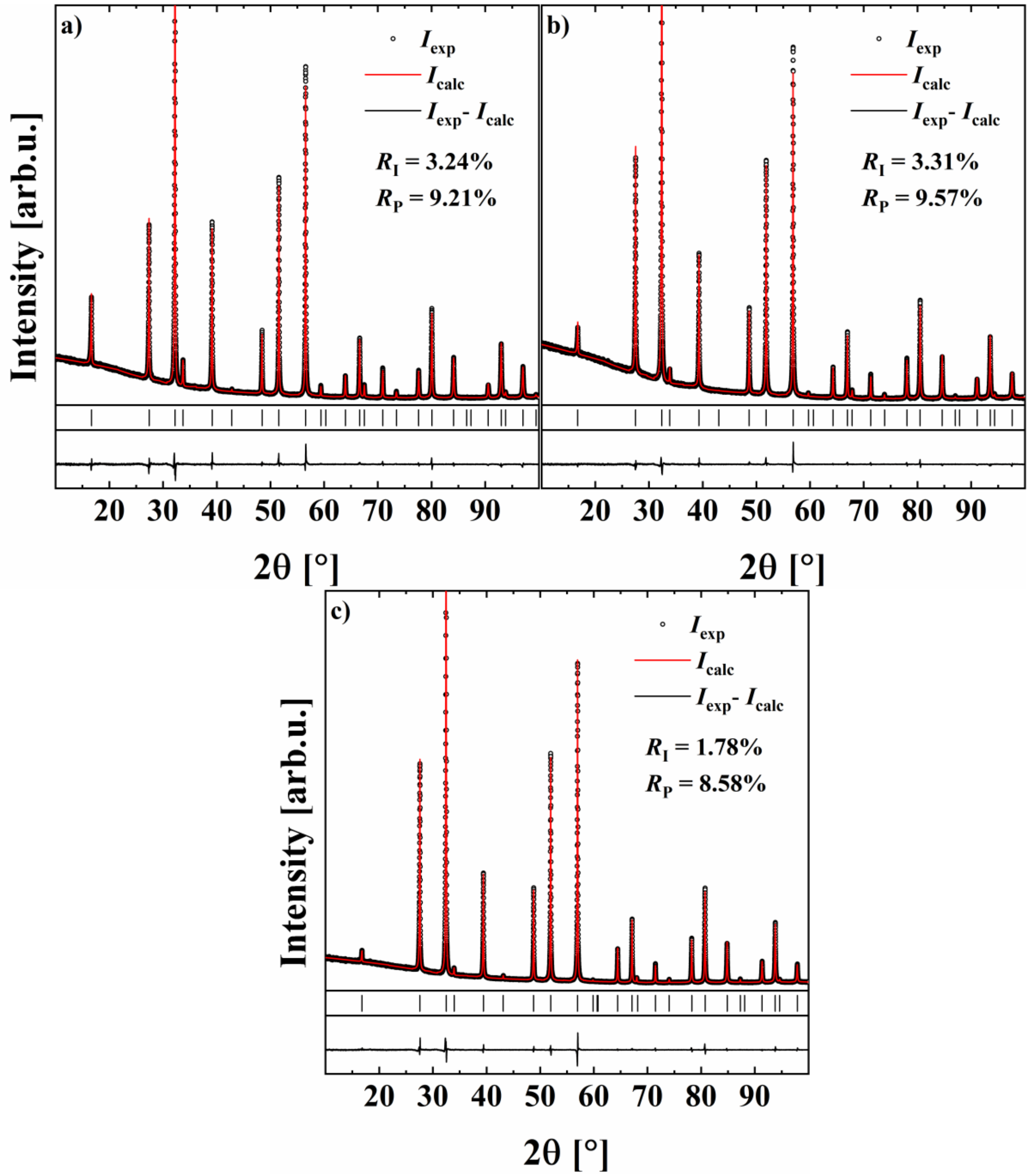

Figure S5. Rietveld refinements of a) $(In_{0.84})[Fe_{0.52}In_{1.48}]S_4$, b) $(In_{0.95})[Fe_{0.85}In_{1.15}]S_4$ and c) $(In)[FeIn]S_4$.

Table S1. Crystallographic data for the $(In_{0.84})[Fe_{0.52}In_{1.48}]S_4$, $(In_{0.95})[Fe_{0.85}In_{1.15}]S_4$ and $(In)[FeIn]S_4$ samples at 293 K obtained from Rietveld refinements.

**Nominal composition**	$\mathbf{(In_{0.84})[Fe_{0.52}In_{1.48}]S_4}$	$\mathbf{(In_{0.95})[Fe_{0.85}In_{1.15}]S_4}$	$\mathbf{(In)[FeIn]S_4}$
Refined Composition	$(In_{0.84})[Fe_{0.46(2)}In_{1.54(2)}]S_4$	$(In_{0.95})[Fe_{0.72(6)}In_{1.28(6)}]S_4$	$(In)[Fe_{1.00(6)}In_{1.00(6)}]S_4$
Space group, no. of formula units Z		$Fd\bar{3}m$ (no. 227), 8	
a /Å	10.6803(3)	10.6314(3)	10.6076(2)
V /Å^3	1218.27(9)	1201.6(1)	1193.59(7)
d /g·cm^{-3}	4.6512(4)	4.6945(4)	4.6048(3)
λ /Å		1.78897	
$2\theta_{max}$ /°; $\sin\theta/\lambda$ (max)		100, 0.428	
No. of refined reflections	32	30	30
R_I, R_P /%	3.24, 9.21	3.31, 9.57	1.78, 8.58
In at 8a ($^1/_8$ $^1/_8$ $^1/_8$), B_{iso}	1.8(1)	1.9(2)	1.7(1)
In/Fe at 16d ($^1/_4$ 0 $^3/_4$), B_{iso}	2.3(1)	2.5(2)	2.2(1)
S at 32e (x x x), x / B_{iso}	0.2574(5) / 2.2(2)	0.2585(7) / 2.2(3)	0.2586(5) / 2.3(2)
SOF (In at 8a)	0.84	0.95	1
SOF (In/Fe at 16d)	0.77(1) / 0.23(1)	0.64(3) / 0.36(3)	0.50(3) / 0.50(3)

Mössbauer spectroscopy

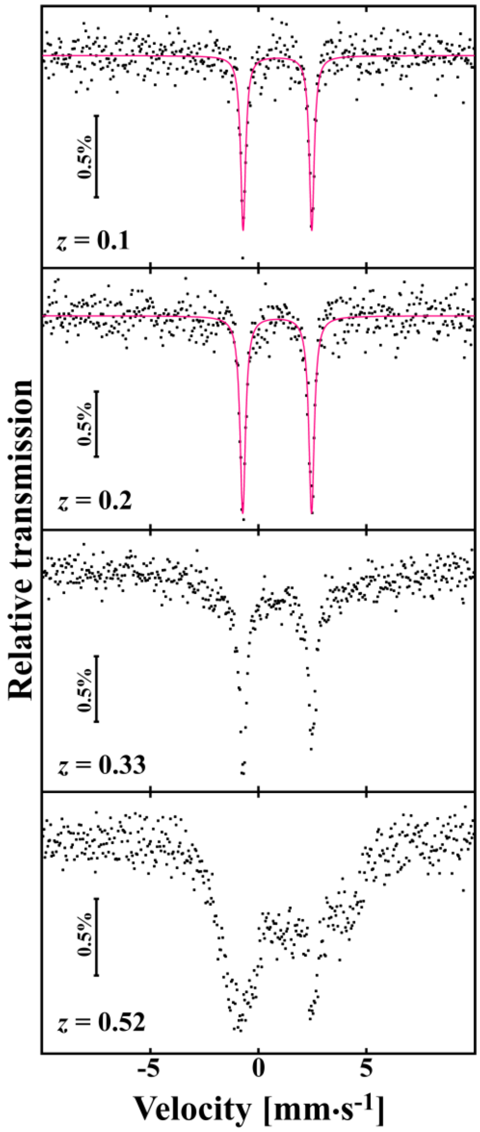

Figure S6. ^{57}Fe Mössbauer spectra of $(In_{0.77})[Fe_{0.1}In_{1.9}]S_4$, $(In_{0.87})[Fe_{0.2}In_{1.8}]S_4$, $(In)[Fe_{0.33}In_{1.67}]S_4$ and $(In_{0.84})[Fe_{0.52}In_{1.48}]S_4$ (from top to bottom). The transmission integral fits are shown in magenta.

Table S2. Fitting parameters of ^{57}Fe Mössbauer spectroscopic measurements at 6 K. δ - isomer shift, ΔE_Q - electric quadrupole splitting, Γ - experimental line width.

**z**	δ **/ mm·s^{-1}**	ΔE_Q **/ mm·s^{-1}**	Γ **/ mm·s^{-1}**
0.1	0.883(5)	3.178(10)	0.273(15)
0.2	0.869(5)	3.185(9)	0.304(13)

Electronic transport properties in the first heating-cooling cycle

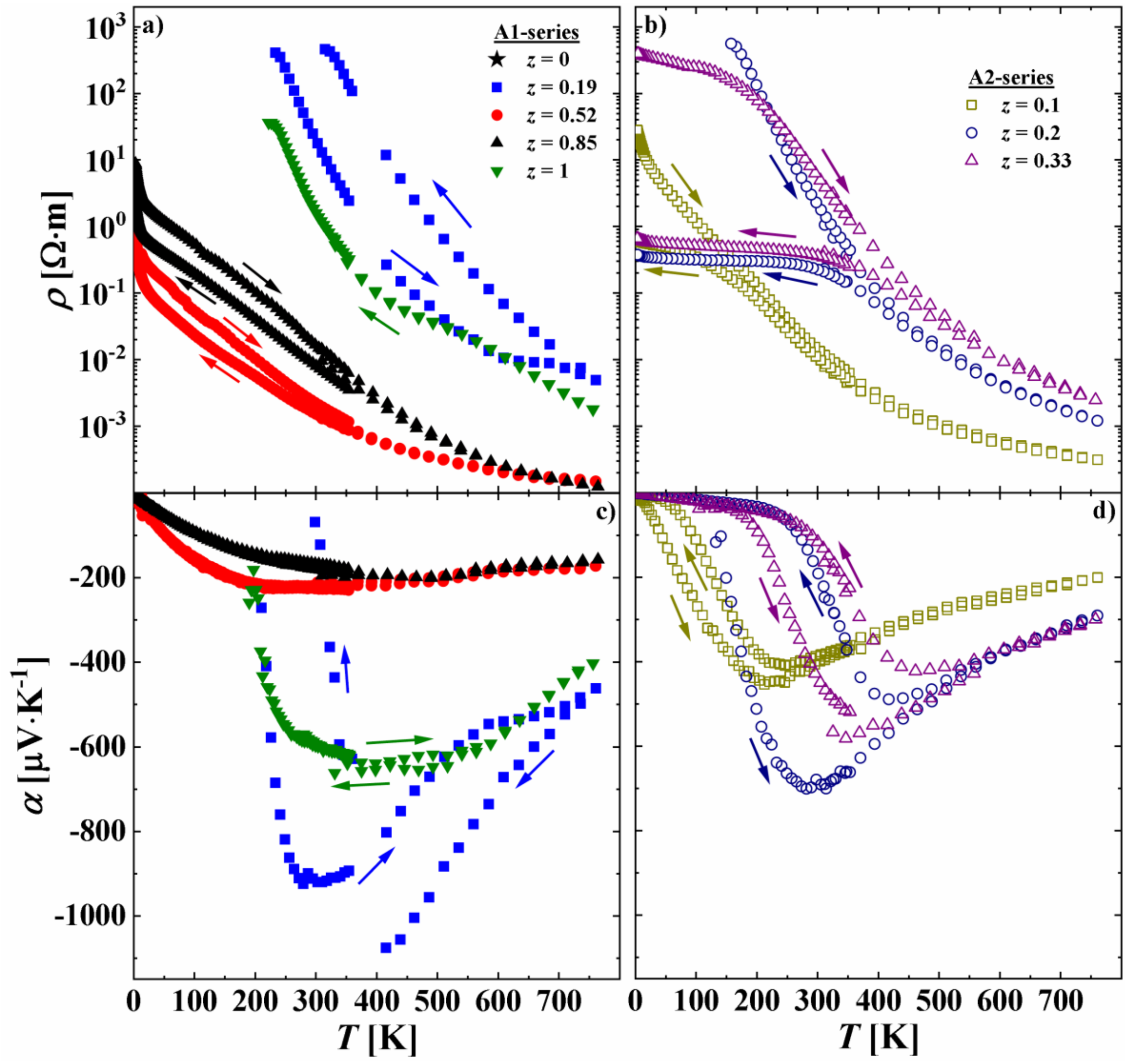

Figure S7. Heating and cooling data of electrical resistivity (a,b) and Seebeck coefficient (c,d) of the A1- and A2-series. For $(In)[FeIn]S_4$, heating and cooling curves above 300 K reproduce each other, while heating data below 200 K were not available.

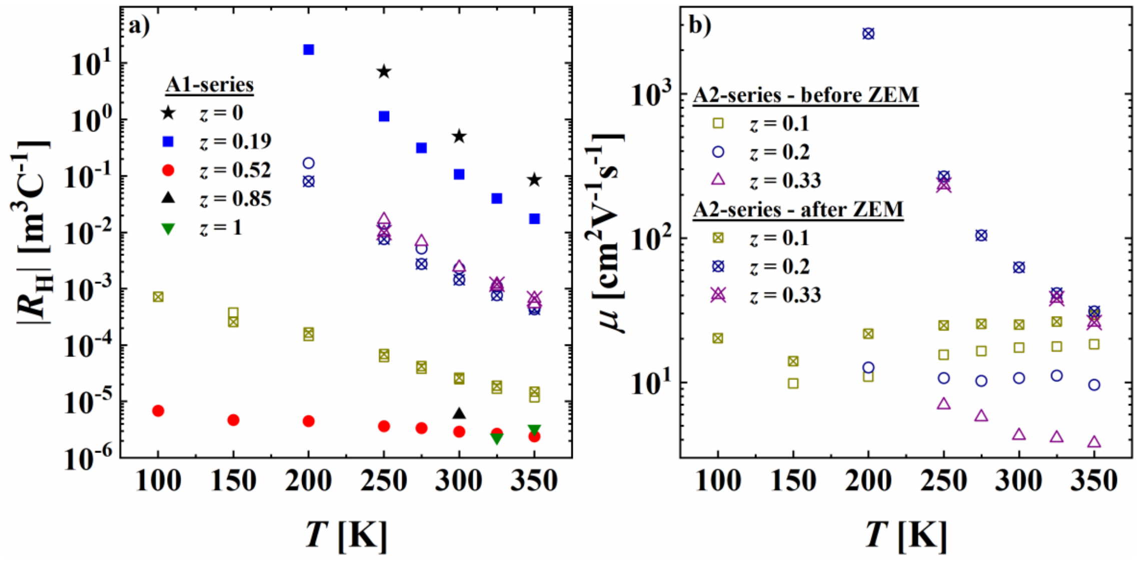

Figure S8. Hall coefficient R_H (a) and charge carrier mobility μ (b) of the A1- and A2-samples.

Analysis of Seebeck coefficient (including small polaron hopping)

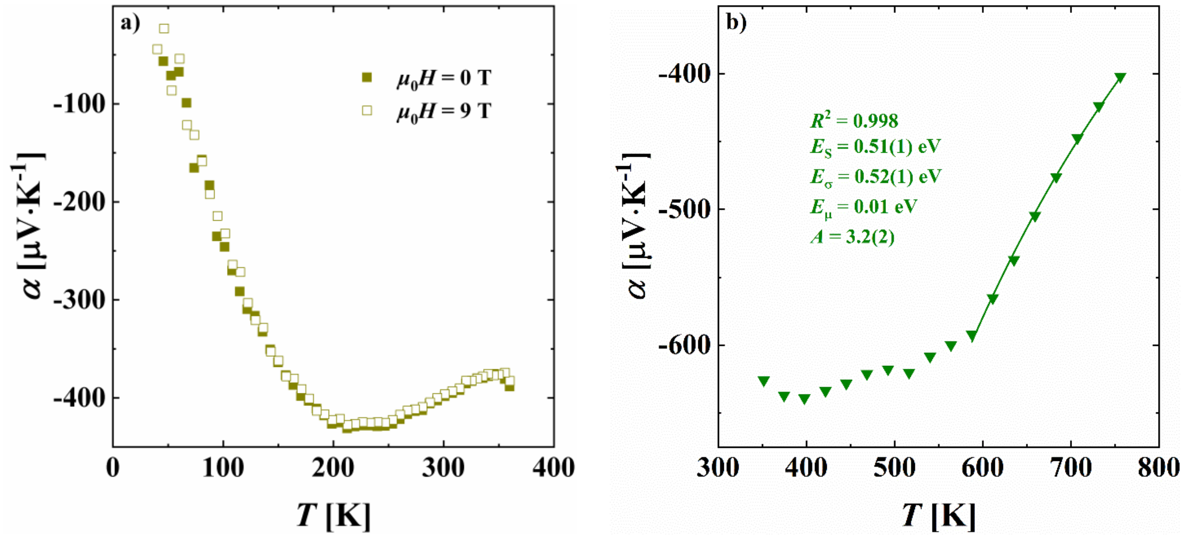

Figure S9. a) Seebeck coefficient α of $(In_{0.77})[Fe_{0.1}In_{1.9}]S_4$ thiospinel, measured without magnetic field and at 9 T. b) Small-polaron hopping model applied to $\alpha(T)$ for $(In)[FeIn]S_4$. Solid lines indicate fits to the equation: $\alpha(T) = -k/e\cdot(E_S/(kT) + A)$. Fitted parameters and the correlation coefficient R^2 are given in the graph.

The HT temperature behavior of the Seebeck coefficient $\alpha(T)$ for a semiconductor can be described with the equation: $\alpha(T) = -k/e\cdot(E_S/(kT) + A)$, where k is the Boltzmann constant in [eV·K^{-1}], $e = 1.602 \times 10^{-19}$ C is the elementary charge, E_S is the carrier-generation activation energy in [eV] and A is the *heat-of-transport* constant, related to the steepness of the electronic density of states $\partial N(E)/\partial E$ of the band.[8-12] In the case of small-polaron hopping (SPH), associated with a narrow energy band, A is typically larger than 2 and E_S is considerably smaller than the conductivity activation energy $E_\sigma = E_a/2$ (half of the activation energy discussed in the main text). For a wide band-gap semiconductor $A \sim 1 - 2$ and $E_S \approx E_\sigma$. The difference between these energies, $E_\mu = E_\sigma - E_S$ is connected with intrinsic hopping of small polarons and can be of the order of ~ 0.1 eV.[9,11] Here, we observe a large $A = 3.2$ (in agreement with SPH) and $E_\mu = 0.01$ eV ≈ 0 (in contradiction with SPH) for $(In)[FeIn]S_4$ (Figure S9b). To shed light on a possible SPH mechanism in $(In)[FeIn]S_4$ additional studies are required.

Single parabolic band model

According to Boltzmann transport theory,[13] the following parameters can be calculated as a function of the reduced Fermi energy η:

Seebeck coefficient

$$\alpha(\eta) = \frac{k}{e}\left[\frac{(r+5/2)F_{(r+3/2)}(\eta)}{(r+3/2)F_{(r+1/2)}(\eta)} - \eta\right]$$

Charge carrier concentration (from the Hall measurement)

$$n(\eta) = \frac{1}{eR_H} = \frac{(2m^*kT)^{3/2}}{3\pi^2\hbar^3}\,\frac{(r+3/2)^2F_{(r+1/2)}^2(\eta)}{(2r+3/2)F_{(2r+1/2)}(\eta)}$$

Fermi integral

$$F_j(\eta) = \int_0^\infty \frac{E^j}{1+e^{(E-\eta)}}\,dE$$

In our calculations we assumed a scattering factor r = 1.5, as for ionized impurity scattering.

References

[1] P. Wyżga, I. Veremchuk, C. Himcinschi, U. Burkhardt, W. Carrillo-Cabrera, M. Bobnar, C. Hennig, A. Leithe-Jasper, J. Kortus, R. Gumeniuk, *Dalton. Trans.* **2019**, *48*, 8350–8360.

[2] P. Wyżga, I. Veremchuk, M. Bobnar, C. Hennig, A. Leithe-Jasper, R. Gumeniuk, *Z. Anorg. Allg. Chem.* **2020**, 10.1002/zaac.202000014, *Accepted*.

[3] I. V. Bodnar, *Semiconductors* **2014**, *48*, 557–561.

[4] N. Barreau, J. C. Bernède, C. Deudon, L. Brohan, S. Marsillac, *J. Cryst. Growth* **2002**, *241*, 4–14.

[5] G. Delgado, A. J. Mora, C. Pineda, T. Tinoco, *Mater. Res. Bull.* **2001**, *36*, 2507–2517.

[6] H. Falcón, P. Tartaj, F. Vaquero, R. M. Navarro, J. L. G. Fierro, J. P. Bolletta, J. M. De Paoli, R. E. Carbonio, M. T. Fernández-Díaz, J. A. Alonso, *Eur. J. Inorg. Chem.* **2016**, 1558–1565.

[7] R. D. Shannon, *Acta Crystallogr. Sect. A* **1976**, *A32*, 751–767.

[8] D. Emin, C. H. Seager, R. K. Quinn, *Phys. Rev. Lett.* **1972**, *28*, 813–816.

[9] A. J. E. Rettie, W. D. Chemelewski, D. Emin, C. B. Mullins, *J. Phys. Chem. Lett.* **2016**, *7*, 471–479.

[10] T. O. Mason, H. K. Bowen, *J. Am. Ceram. Soc.* **1981**, *64*, 237–242.

[11] R. Metselaar, R. E. J. Van Tol, P. Piercy, *J. Solid State Chem.* **1981**, *38*, 335–341.

[12] M. A. Ahmed, M. K. El-Nimr, A. Tawfik, A. M. El-Hasab, *Phys. Status Solidi* **1991**, *123*, 501–506.

[13] H. J. Goldsmid, *Introduction to Thermoelectricity*; Springer-Verlag Berlin Heidelberg, **2010**.
